# Supplementary material for: Specific Nucleic AcId Ligation for the detection of Schistosomes: SNAILS
Source: PLoS Negl Trop Dis. 2022 Jul 26;16(7):e0010632. doi: 10.1371/journal.pntd.0010632 (PMC9355235; doi:10.1371/journal.pntd.0010632)
Supplement: S1 Text — (DOCX) [file pntd.0010632.s001.docx]

**S1 Text**

**Specific Nucleic AcId Ligation for the detection of Schistosomes: SNAILS**

Alexander James Webb^1#^, Fiona Allan^2#^, Richard J. R. Kelwick^1#^, Feleke Zewge Beshah^3^, Safari Methusela Kinung’hi^4^, Michael R. Templeton^5^, Aidan Mark Emery^2*^, and Paul S. Freemont^1,6,7*^

^1^Section of Structural and Synthetic biology, Department of Infectious Disease, Imperial College London, London, UK. ^2^Department of Life Sciences, Natural History Museum, London, UK. ^3^College of Natural and Computational Sciences, Addis Ababa University, Arat Kilo, Addis Ababa, Ethiopia. ^4^National Institute of Medical Research (NIMR), Mwanza Center, Mwanza, Tanzania. ^5^Department of Civil and Environmental Engineering, Imperial College London, London, UK. ^6^The London Biofoundry, Imperial College Translation and Innovation Hub, White City Campus, London, UK. ^7^UK Dementia Research Institute Care Research and Technology Centre, Imperial College London, Hammersmith Campus, London, UK.

^#^Joint first authors.

^*^[p.freemont@imperial.ac.uk](mailto:p.freemont@imperial.ac.uk) (PF)

[a.emery@nhm.ac.uk](mailto:a.emery@nhm.ac.uk) (AE)

**
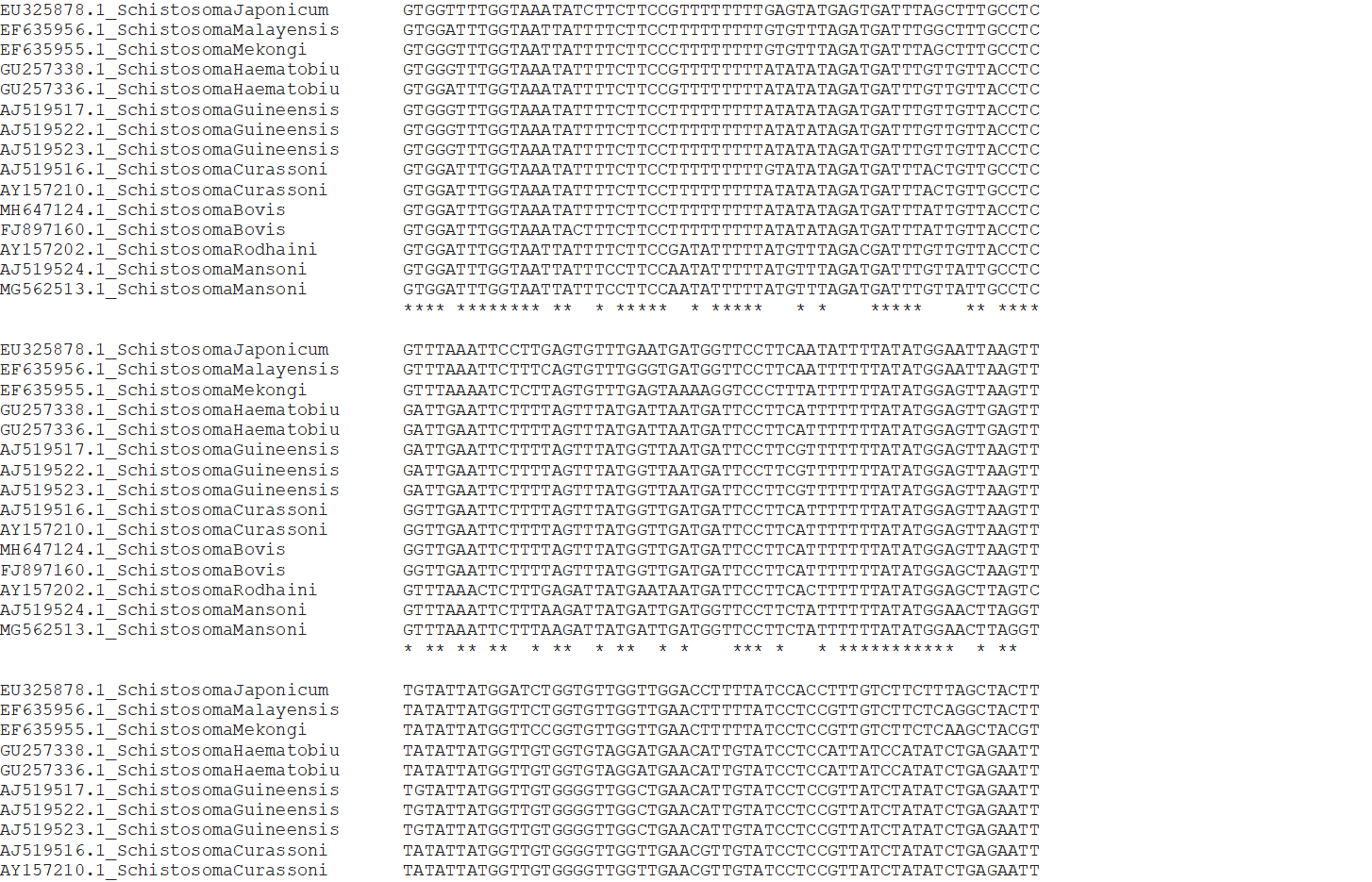

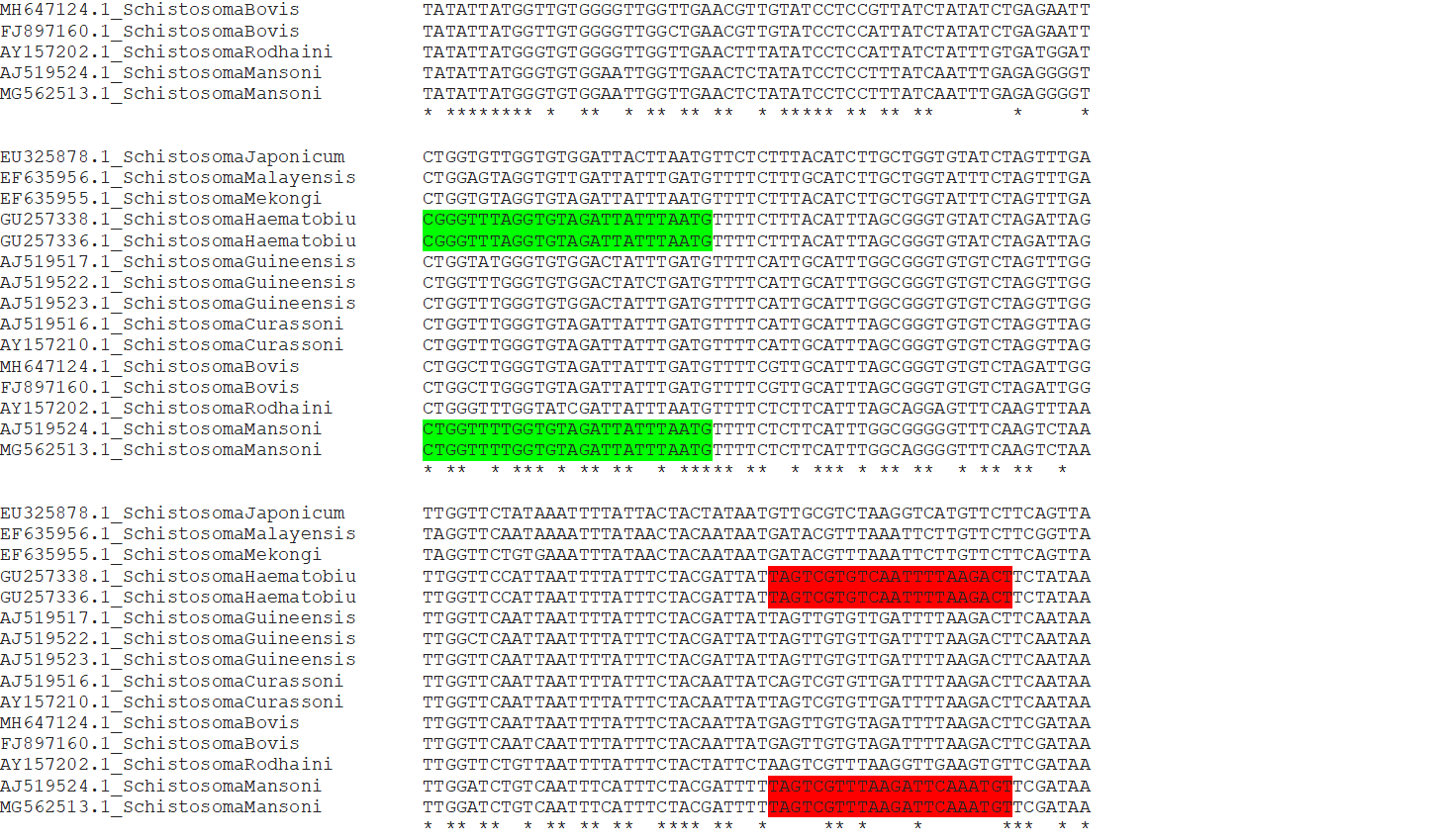
**

**
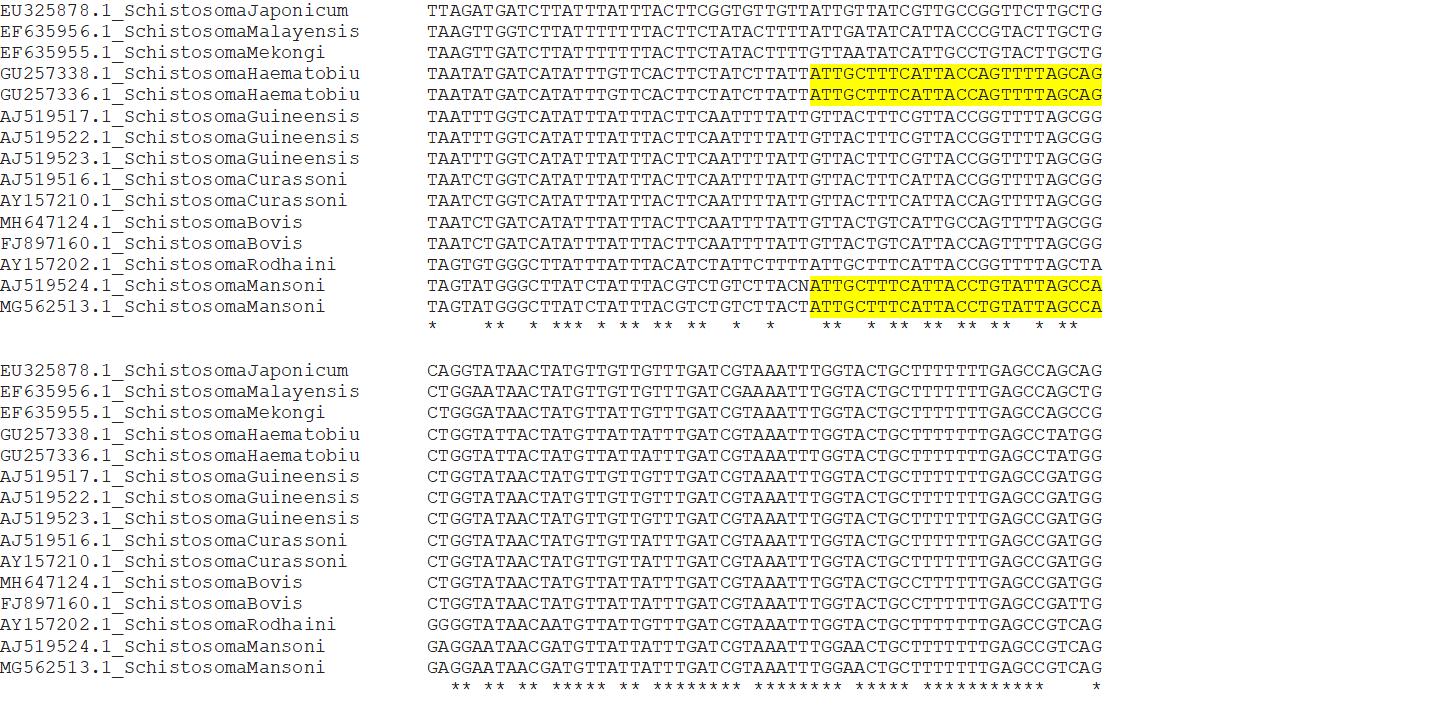

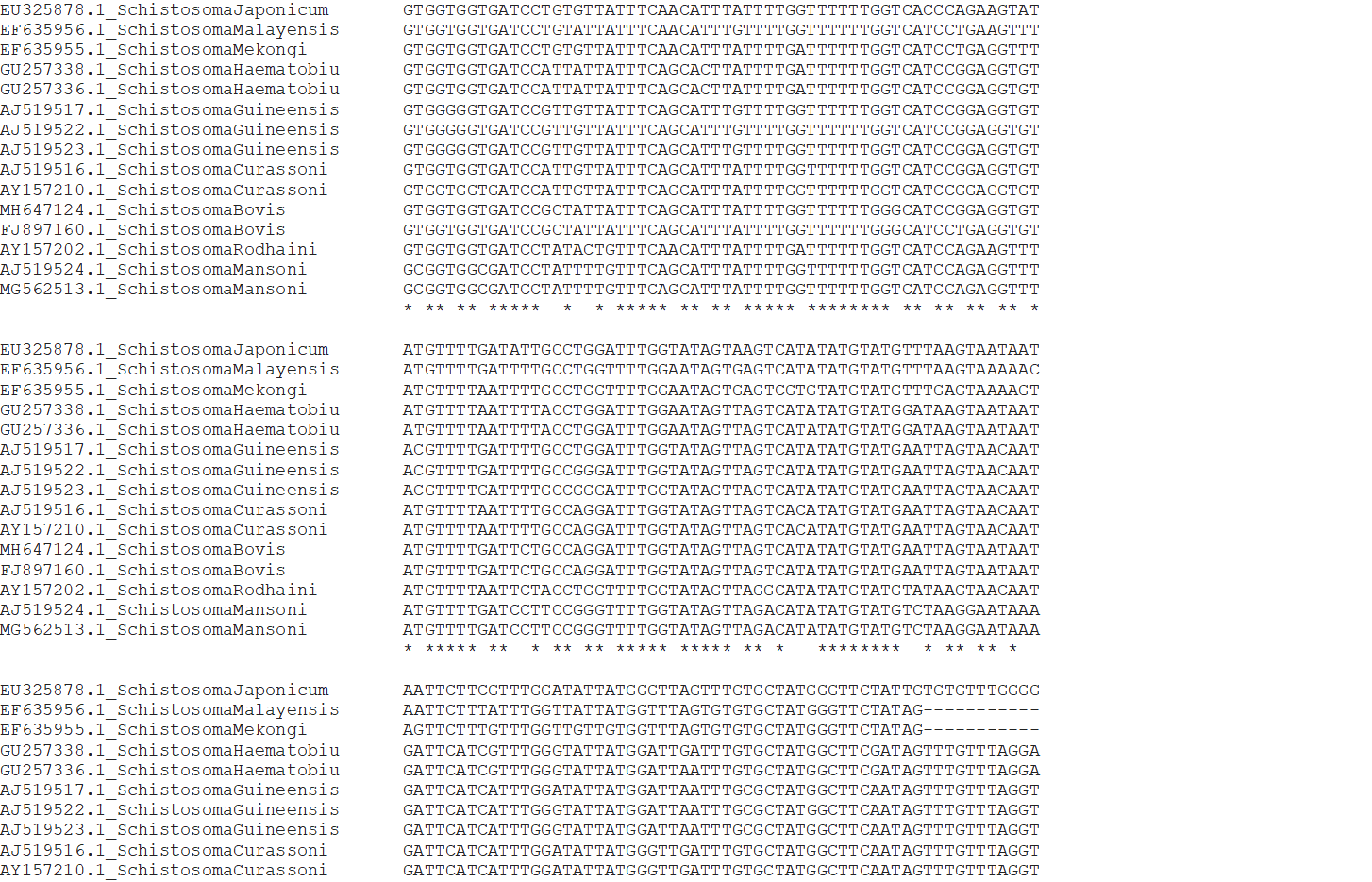
**

**
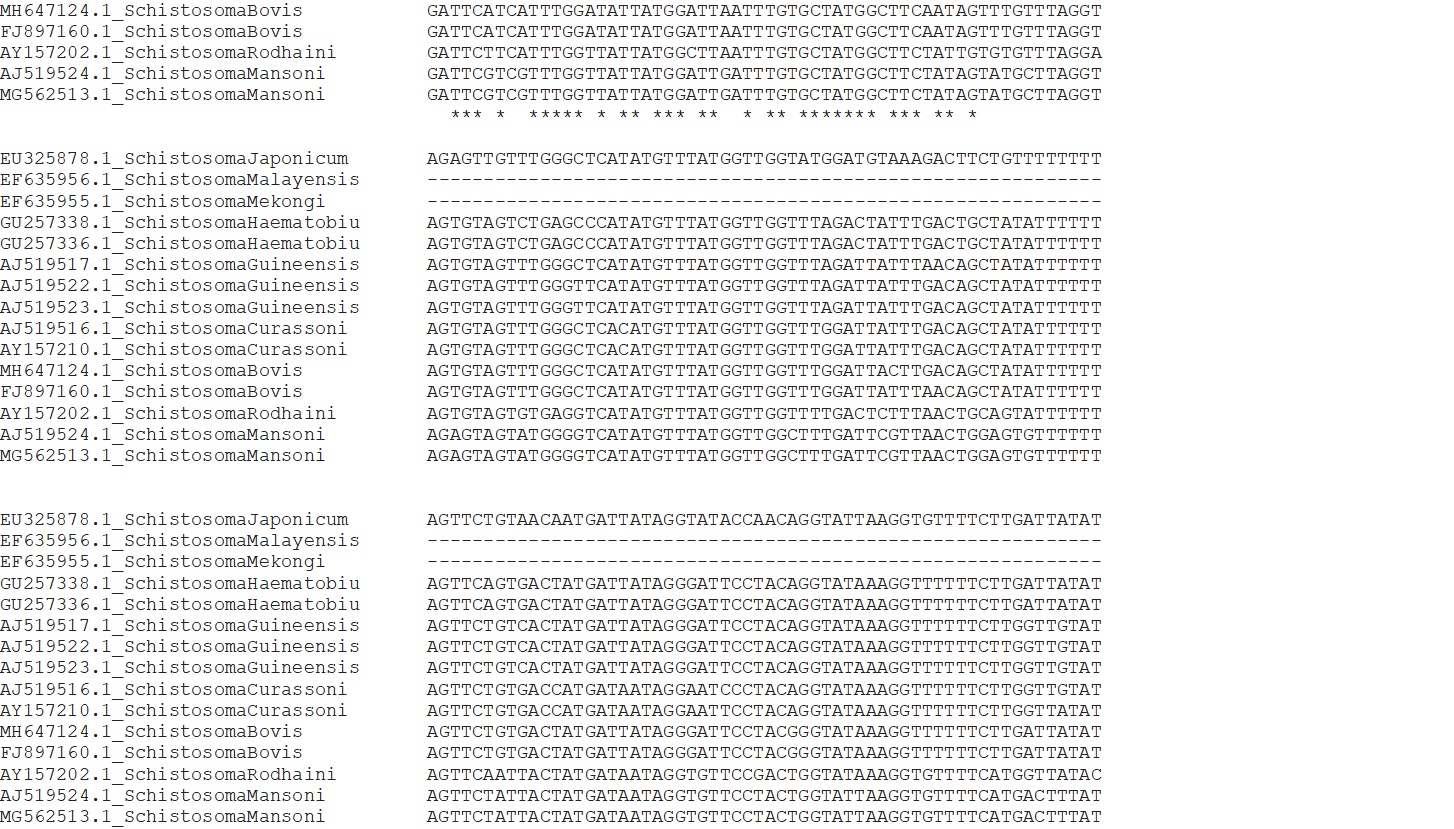

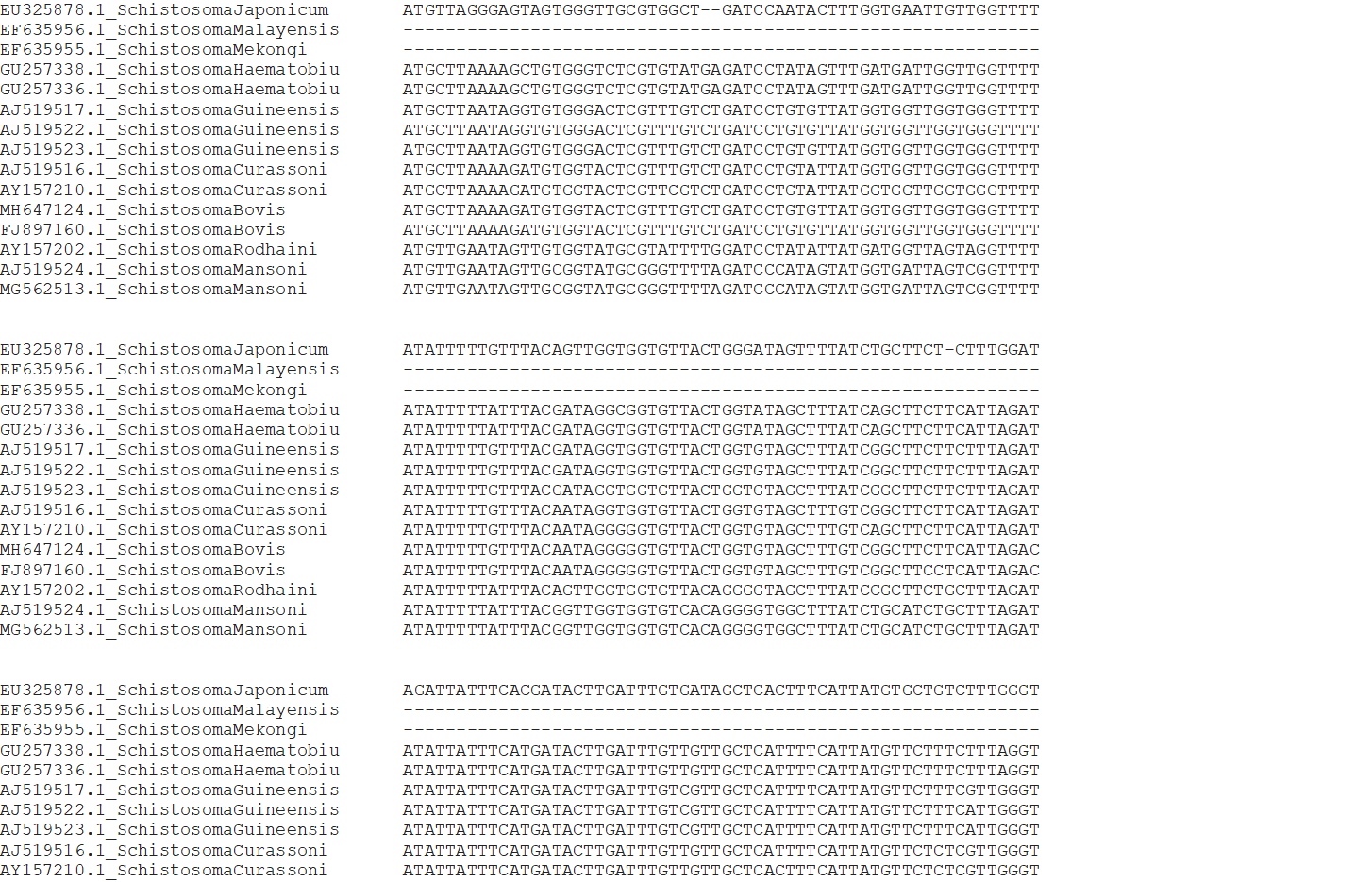
**

**
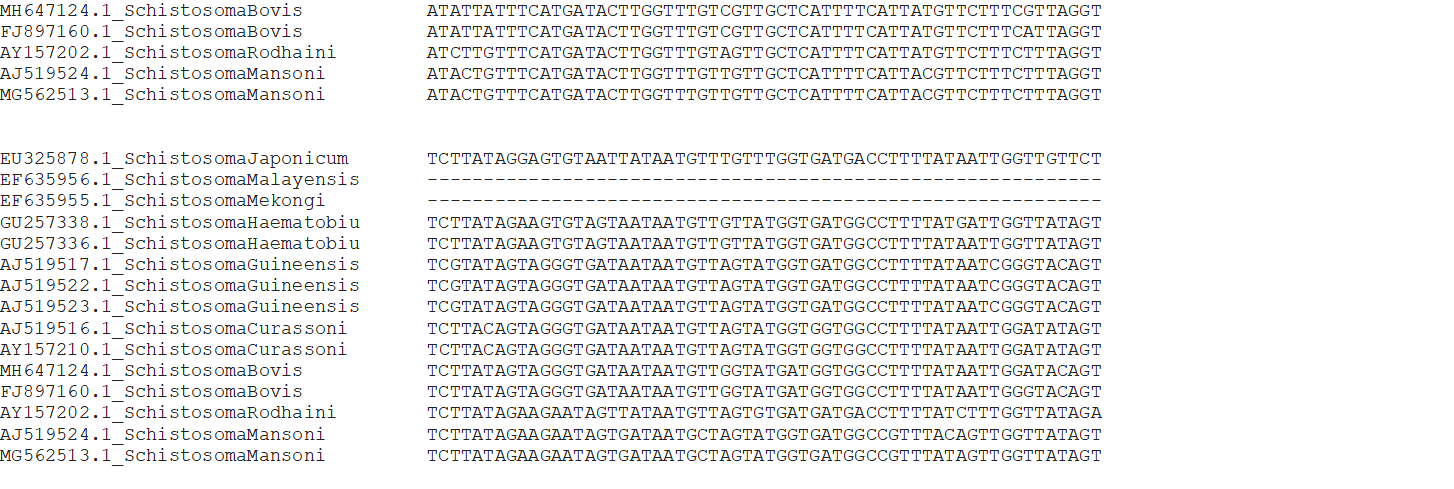

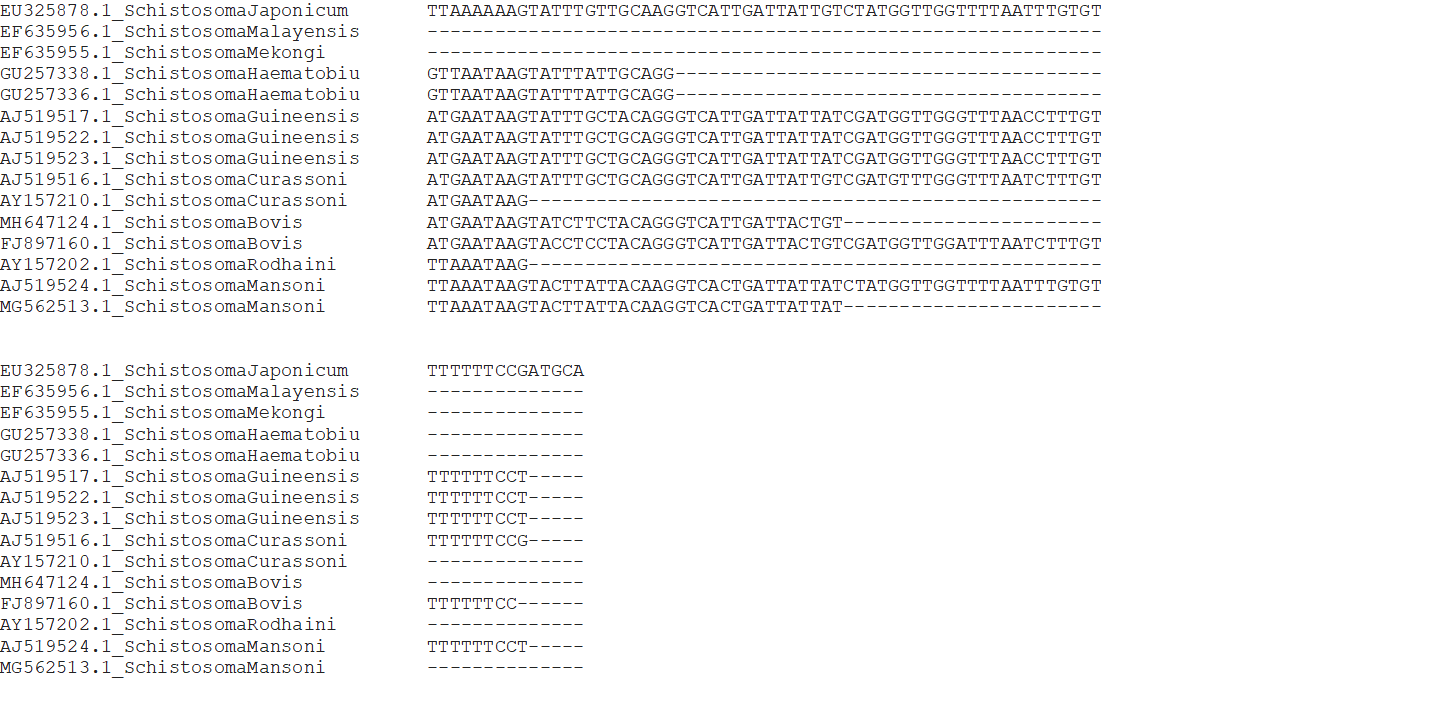
**

**Figure A in S1 Text. MUSCLE alignment of *cox*1 gene sequences from selected schistosome species.** Nucleotide sequences accessed from GenBank and aligned here, are detailed in Table B in S1 Text. Sequences were aligned using MUSCLE[1] with default parameters. 5’ (green text) and 3’ (yellow text) primer binding sites for the amplification of the 180 base target regions, as well as the 22-base biosensor target regions (red text) are indicated for both *S. mansoni* and *S. haematobium*.

**
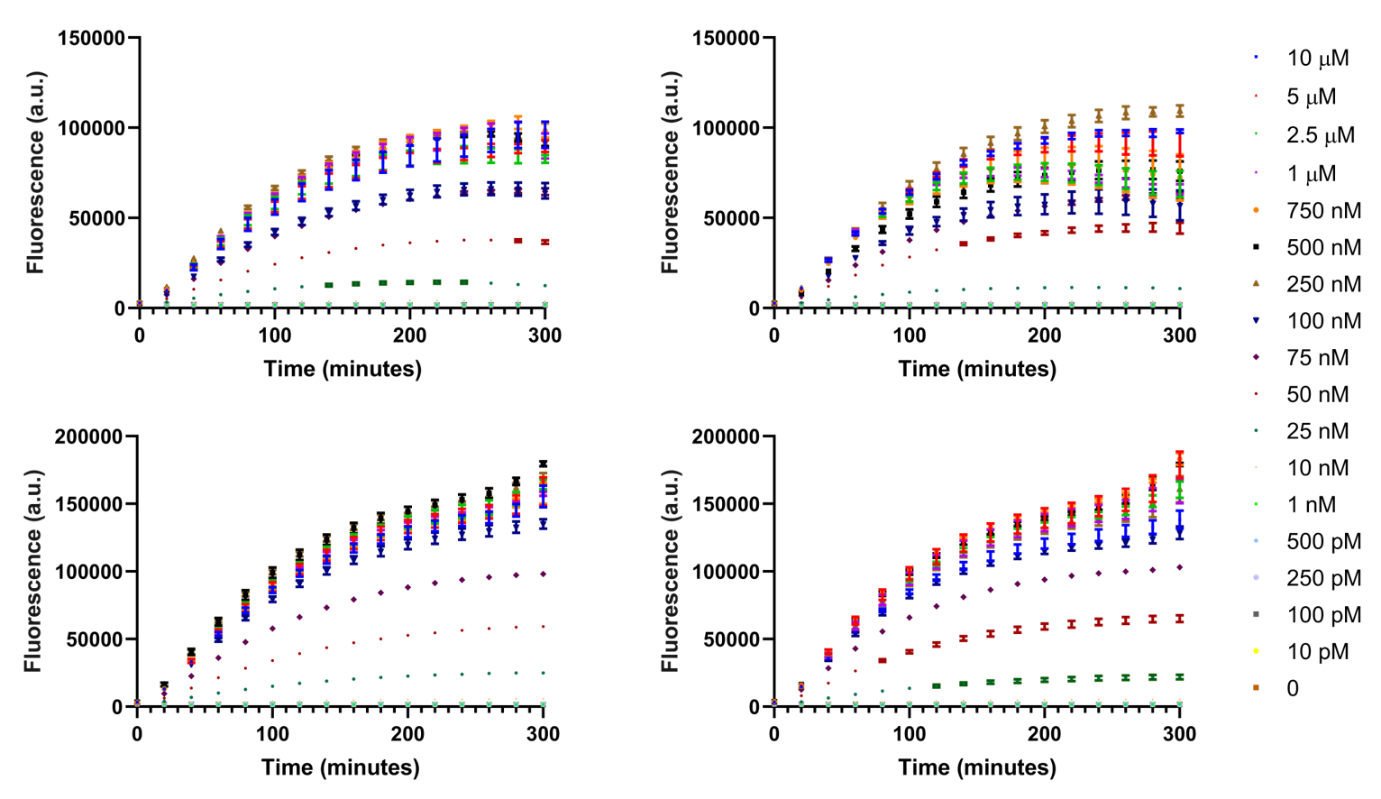
**

**Figure B in S1 Text. Sensitivity of *S. mansoni* probe set 1.** The sensitivity of *S. mansoni* probe set 1 (SM_A1/SM_B1) against a range of concentrations of the 22-base *S. mansoni* target (SM_WT, AJW791) were tested. Probe concentrations were set at 200 nM of each half probe. The concentrations of target DNA tested are indicated in the key. Four reaction set runs are shown separately, with *n=*3 per graph (1 replicate per reaction, each reaction split into triplicate runs). Error bars denote standard error of the mean.

**
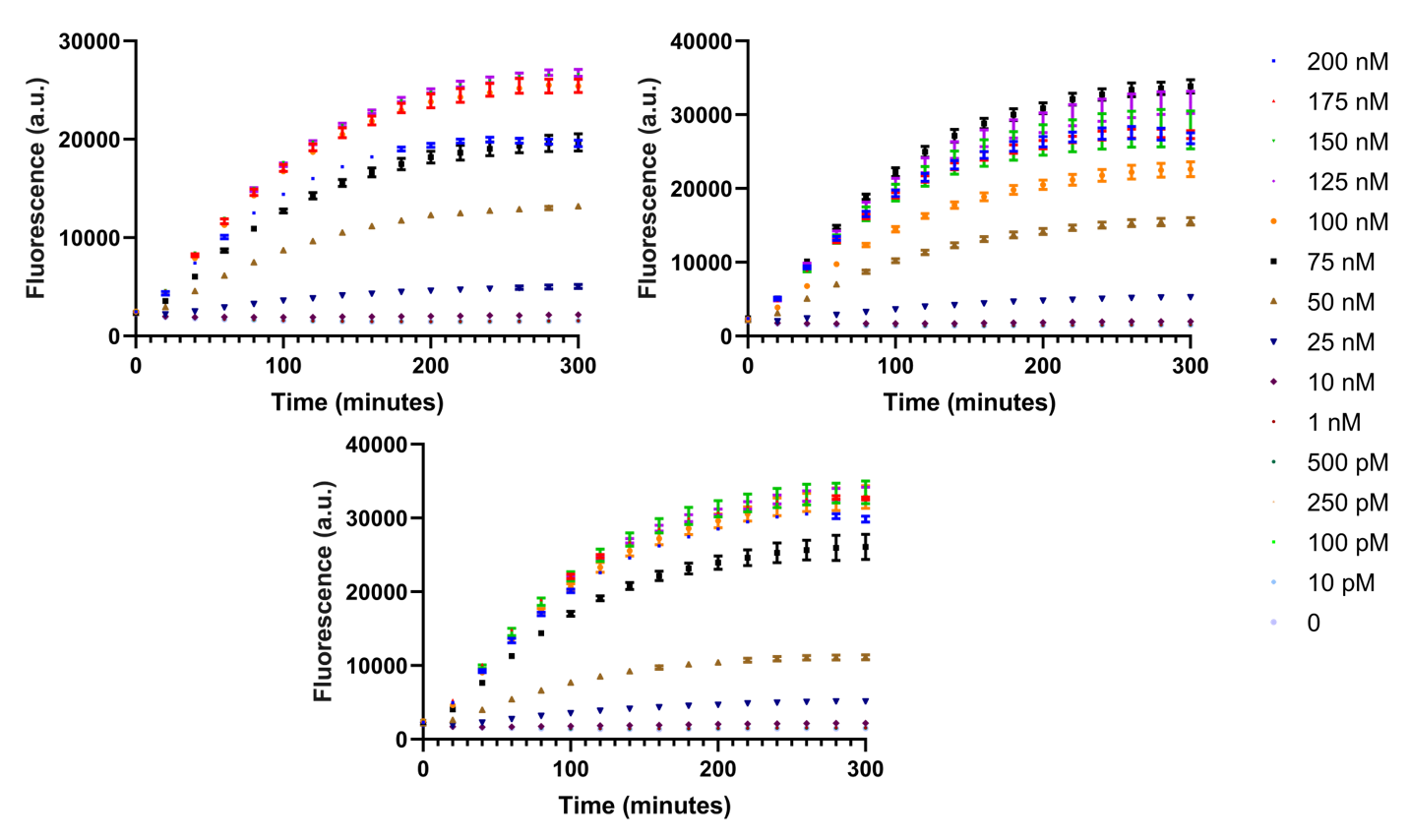
**

**Figure C in S1 Text. Determination of probe concentration to give a measurable output.** Concentration of probes required to detect 50 nM of *S. mansoni* 22-base target (SM_WT, AJW791). The concentrations of both probe halves tested are indicated in the key. Three reaction set runs are shown separately, with *n*=3 per graph (1 replicate per reaction, each reaction split into triplicate runs). Error bars denote standard error of the mean.

**
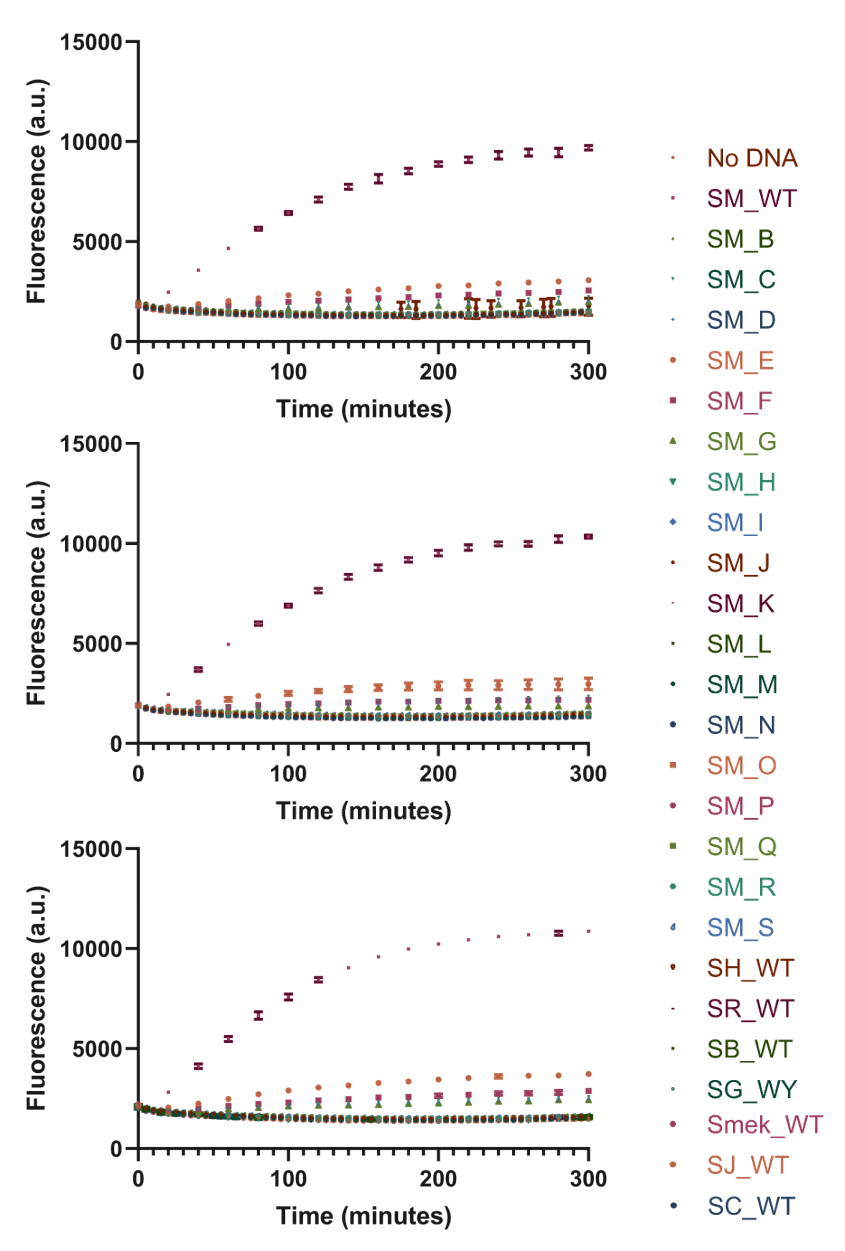
**

**Figure D in S1 Text. Specificity of *S. mansoni* probe set 1 against a range of DNA targets.** Both half probes and the target DNA concentrations were tested at 50 nM. Targets are listed in the key, and further details of these targets are supplied in Table B in S1 Text. Three reaction set runs are shown separately, with *n*=3 per graph (1 replicate per reaction, each reaction split into triplicate runs). Error bars denote standard error of the mean.

**
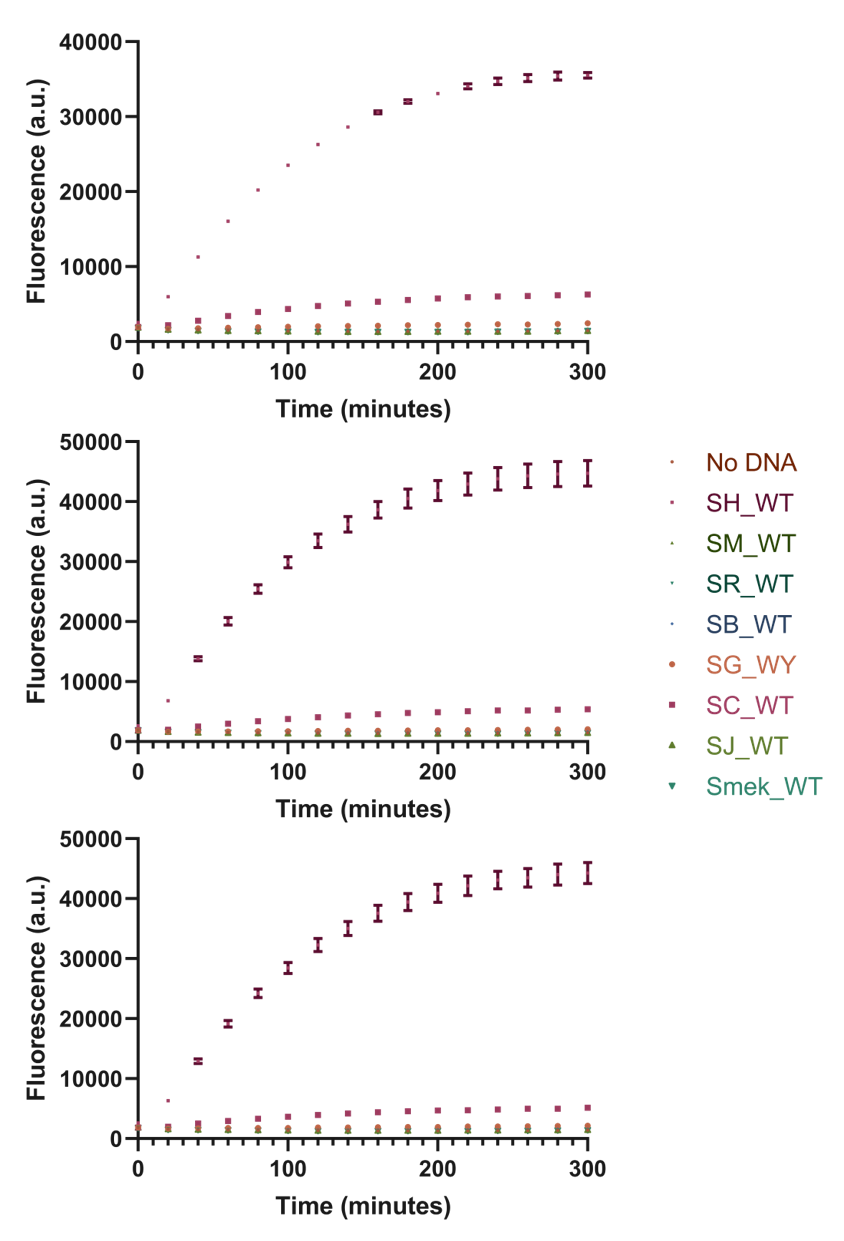
**

**Figure E in S1 Text. Specificity of *S. haematobium* probe set 1 against a range of DNA targets.** Both half probes and the target DNA concentrations were tested at 50 nM. Targets are listed in the key, and further details of these targets are supplied in Table B in S1 Text. Three reaction set runs are shown separately, with *n*=3 per graph (1 replicate per reaction, each reaction split into triplicate runs). Error bars denote standard error of the mean.

**
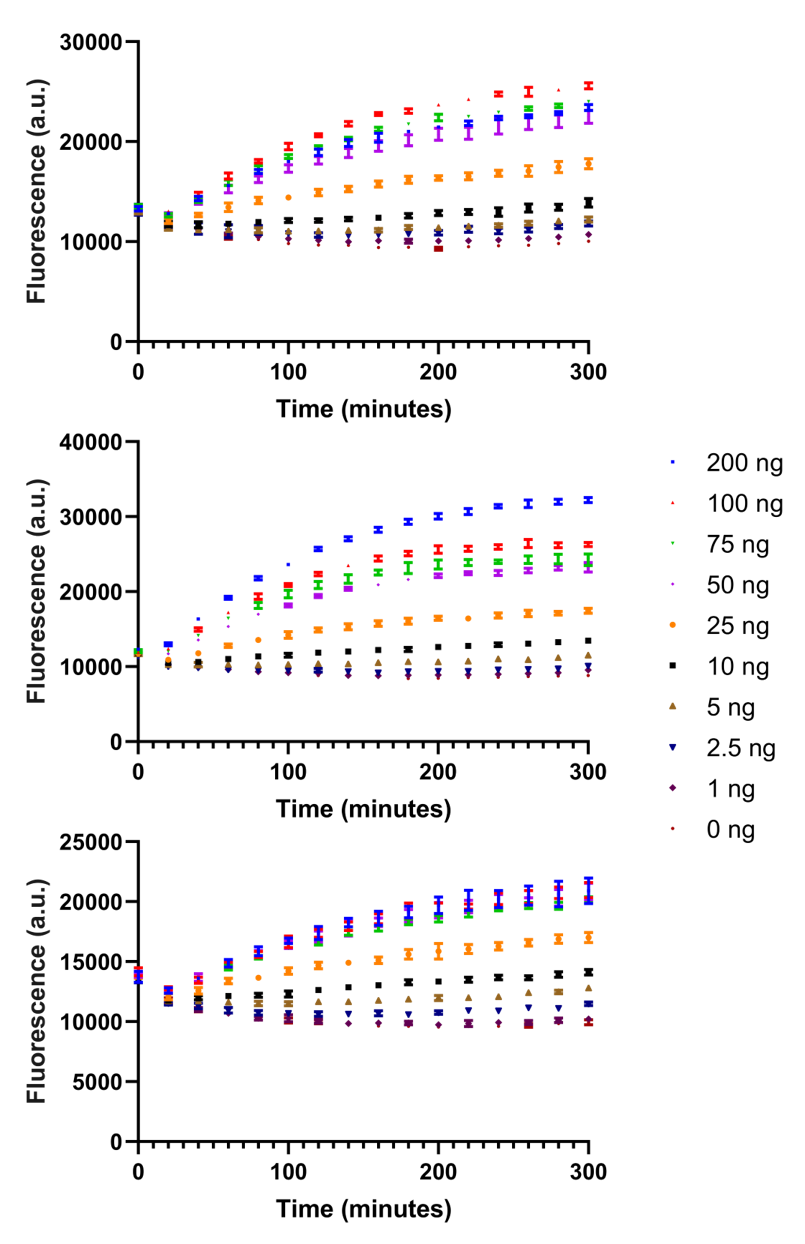
**

**Figure F in S1 Text. *S. mansoni* probe set 1 can detect the synthetic *S. mansoni*-specific 180-base target.** Both half probes were tested at 50 nM, while the amount (ng) of synthetic *S. mansoni*-specific 180-base target (SM_180, AJW874) tested are indicated in the key. Three reaction set runs are shown separately, with *n*=3 per graph (1 replicate per reaction, each reaction split into triplicate runs). Error bars denote standard error of the mean.

**
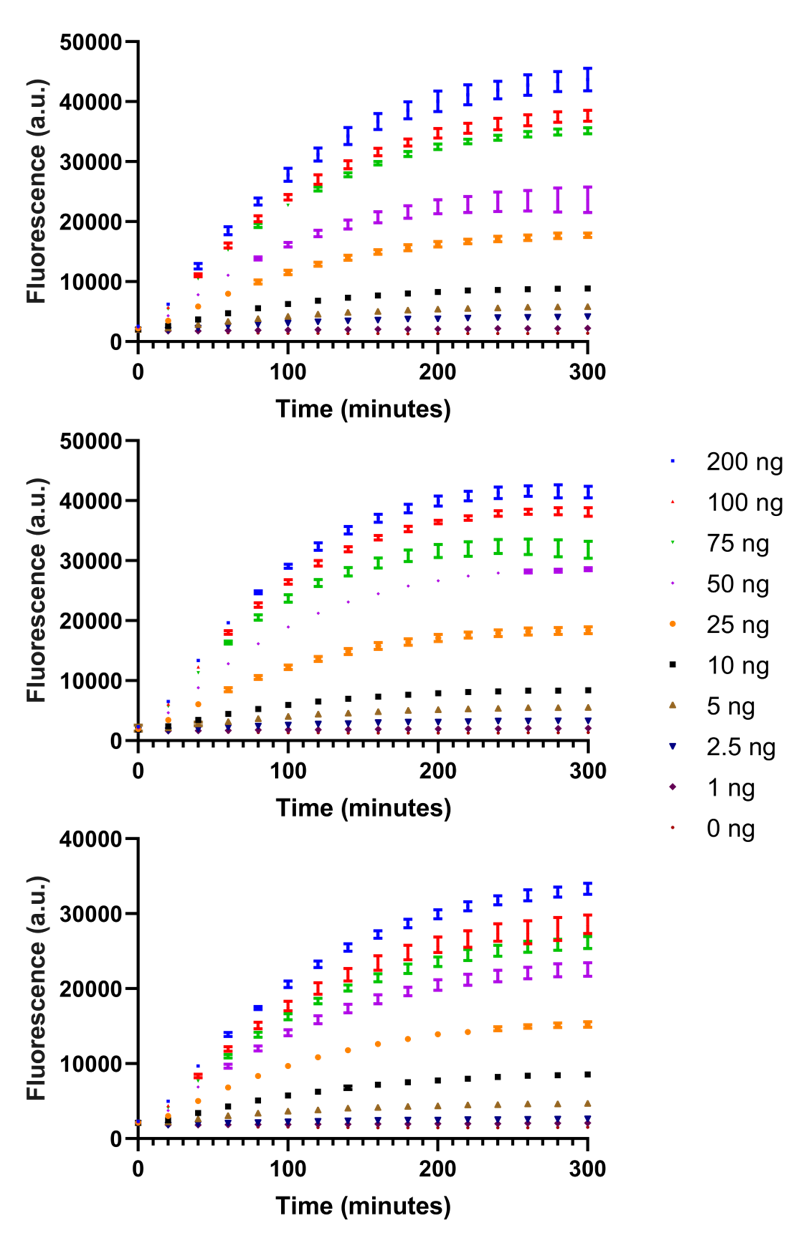
**

**Figure G in S1 Text. *S. haematobium* probe set 1 can detect the synthetic *S. haematobium*-specific 180-base target.** Both half probes were tested at 50 nM, while the amount (ng) of synthetic *S. haematobium*-specific 180-base target (SH_180, AJW875) tested are indicated in the key. Three reaction set runs are shown separately, with *n*=3 per graph (1 replicate per reaction, each reaction split into triplicate runs). Error bars denote standard error of the mean.

**
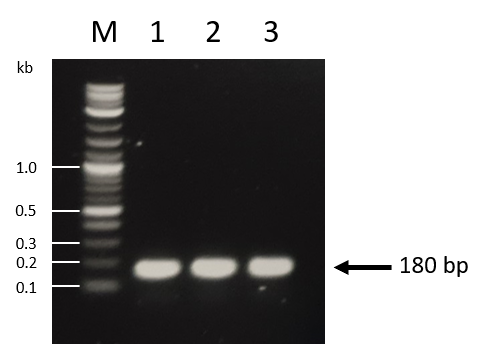
**

**Figure H in S 1 Text. Validation of PCR amplification step.** *S. mansoni* target regions from plasmids or adult worm gDNA were PCR amplified with primer pair 5-SM-PCR/3-SM-PCR. Products amplified are 180 bp in length. Lane 1 (M) is the Quick-load 1 kb plus DNA ladder (N0469S, New England Biolabs, USA), lanes 2-4 (1-3) represent three separate PCR reactions. Size of the bands from the ladder are indicated on the left in kb. Bands were separated on a 1.5% agarose gel in 1X Tris-acetate-EDTA buffer for 30 minutes at 100 volts. Bands on the agarose gel were visualised using the Gel Doc XR+ system with Image Lab software (model # Universal Hood II; Bio-Rad Laboratories Inc., USA).


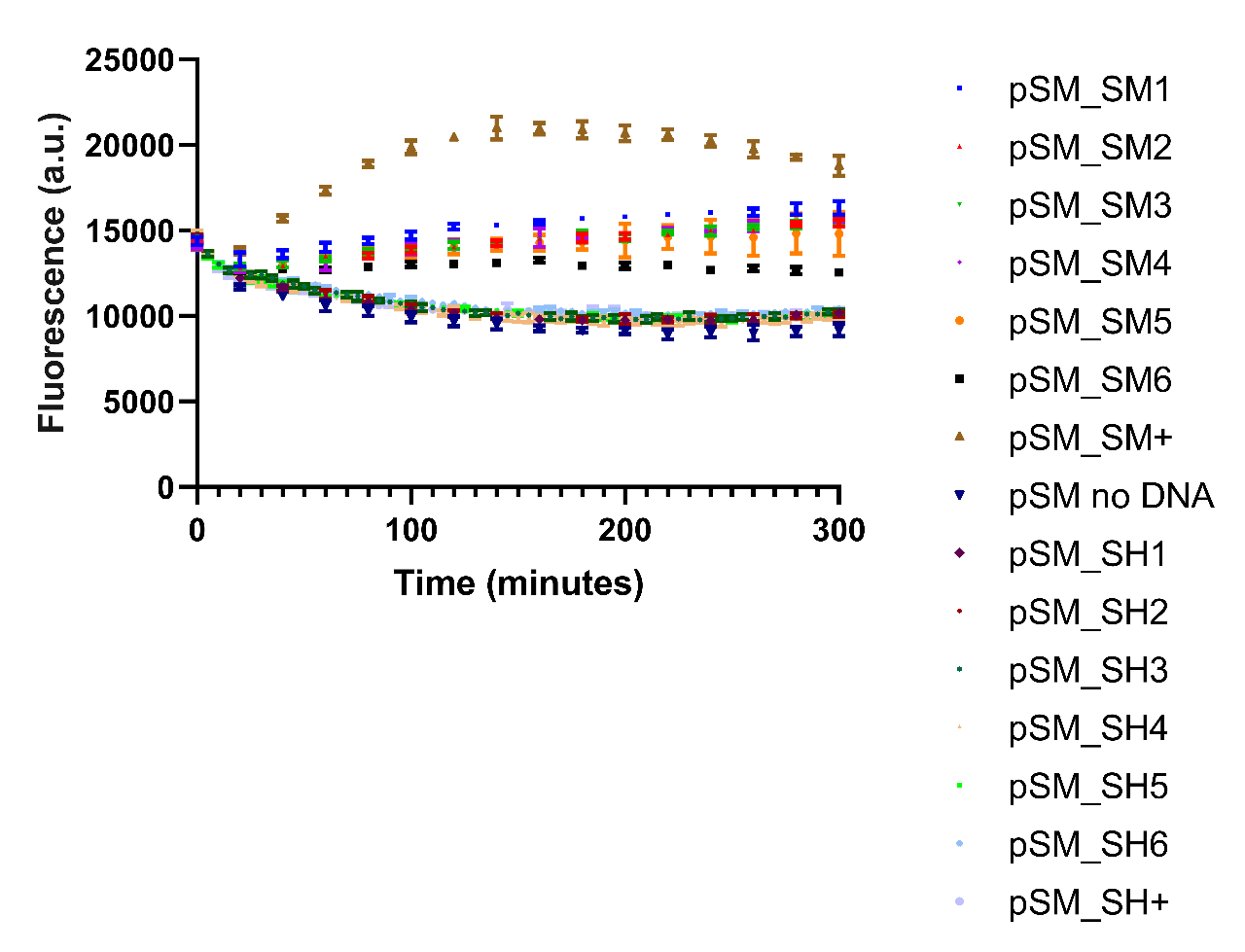


**Figure I in S1 Text. *S. mansoni* probe set 1 recognition of ssDNA derived from plasmid DNA.** The 180-base target regions were PCR amplified from plasmids pAJW250 (*S. mansoni* insert DNA) and pAJW251 (*S. haematobium* insert DNA) and treated as described in main paper text to produce purified ssDNA. Six PCR reactions per plasmid sample were tested against the probe set. 50 nM of each probe half was incubated with 30 ng of target ssDNA. As controls, the probe pair was also incubated against 30 ng of the synthetic targets SM_180 and SH_180. Reactions are identified as follows: pSM_SM1-6 (*S. mansoni* probes against *S. mansoni* plasmid derived ssDNA), pSM_SM+ (*S. mansoni* probes against *S. mansoni* synthetic target), pSM_SH1-6 (*S. mansoni* probes against *S. haematobium* plasmid derived ssDNA), pSM_SH+ (*S. mansoni* probes against *S. haematobium* synthetic target). *n* = 3 (1 replicate per each reaction split into triplicate runs). Error bars denote standard error of the mean.


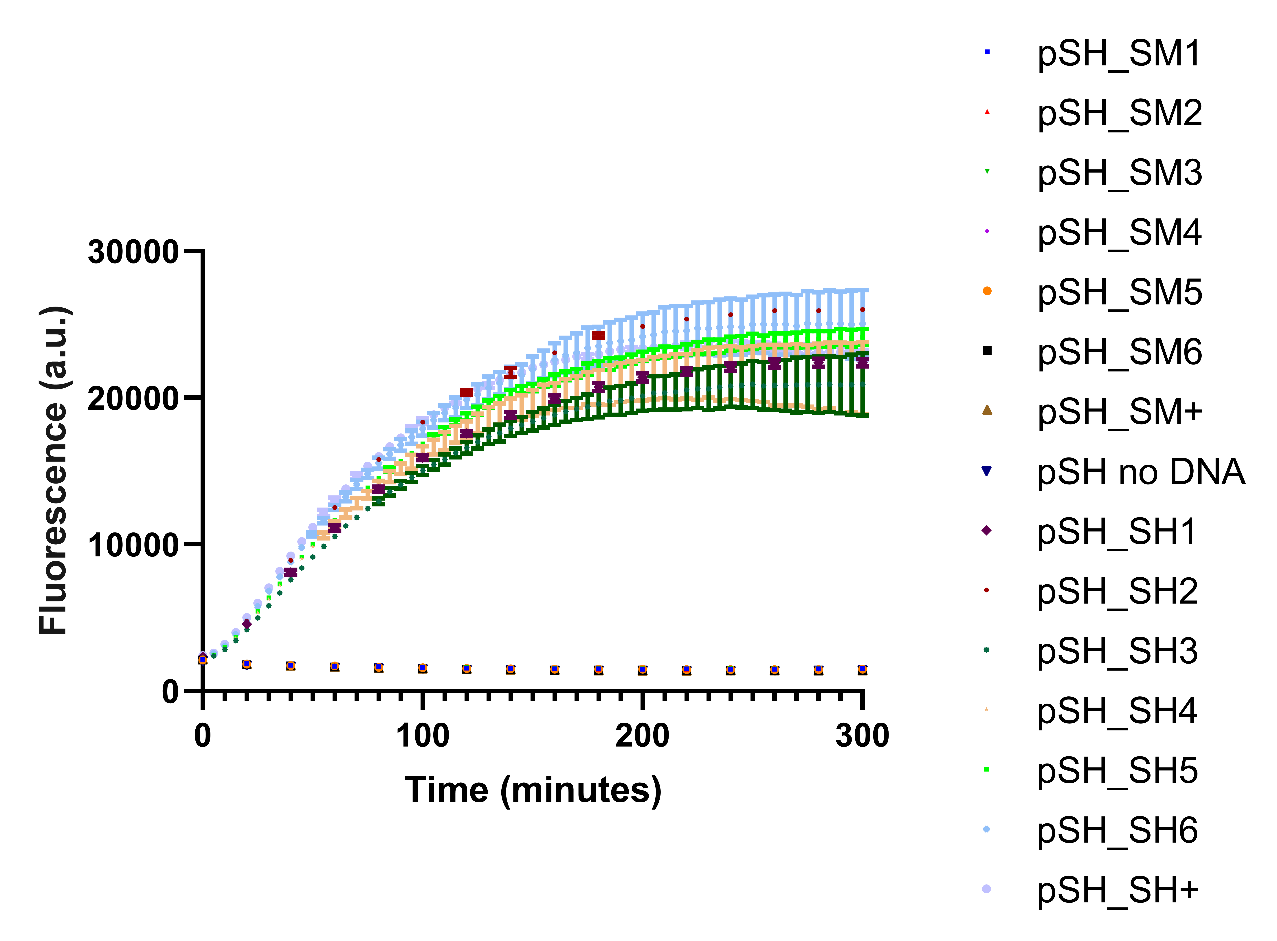


**Figure J in S1 Text. *S. haematobium* probe set 1 recognition of ssDNA derived from plasmid DNA.** The 180-base target regions were PCR amplified from plasmids pAJW250 (*S. mansoni* insert DNA) and pAJW251 (*S. haematobium* insert DNA) and treated as described in main paper text to produce purified ssDNA. Six PCR reactions per plasmid sample were tested against the probe set. 50 nM of each probe half was incubated with 30 ng of target ssDNA. As controls, the probe pair was also incubated against 30 ng of the synthetic targets SM_180 and SH_180. Reactions are identified as follows: pSH_SM1-6 (*S. haematobium* probes against *S. mansoni* plasmid derived ssDNA), pSH_SM+ (*S. haematobium* probes against *S. mansoni* synthetic target), pSH_SH1-6 (*S. haematobium* probes against *S. haematobium* plasmid derived ssDNA), pSH_SH+ (*S. haematobium* probes against *S. haematobium* synthetic target). *n* = 3 (1 replicate per each reaction split into triplicate runs). Error bars denote standard error of the mean.

**
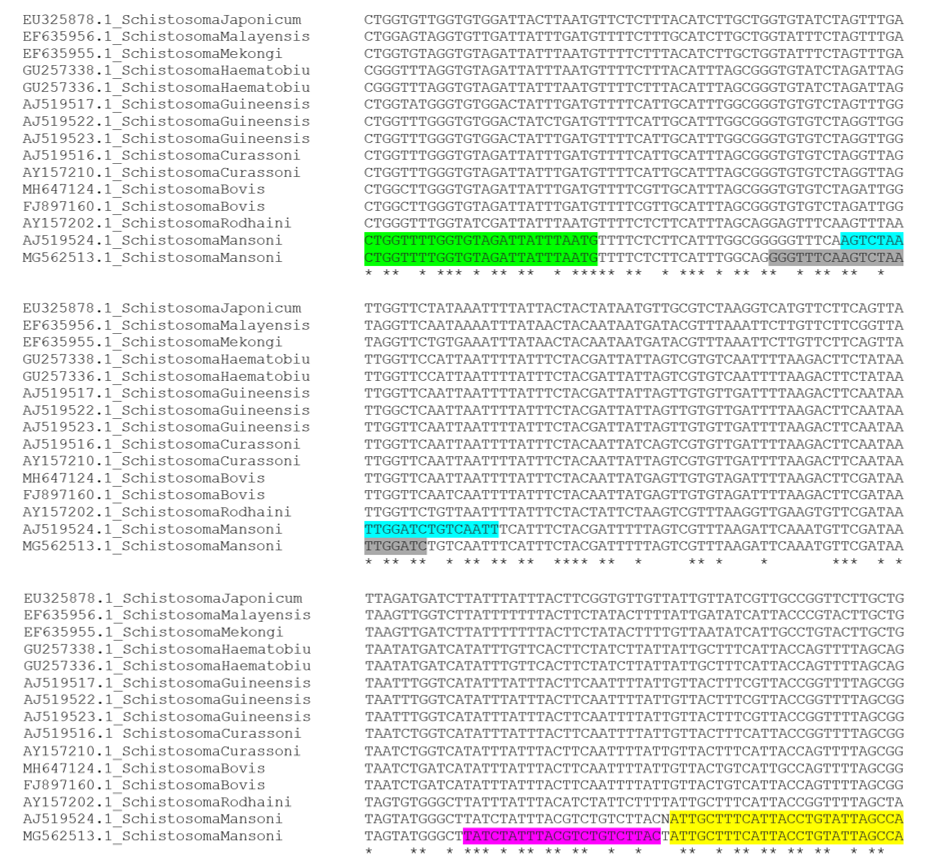
**

**Figure K in S1 Text. Location of new *S. mansoni*-specific probe designs in the 180-base target region.** Nucleotide sequences accessed from GenBank and aligned here, are detailed in Table B in S1 Text. Sequences were aligned using MUSCLE[1] with default parameters. 5’ (green text) and 3’ (yellow text) primer binding sites for the amplification of the 180-base target regions, as well as the 22-base biosensor target regions for probe 2 (grey text), probe 3 (light blue text) and probe 5 (purple text) are indicated for *S. mansoni*.

**
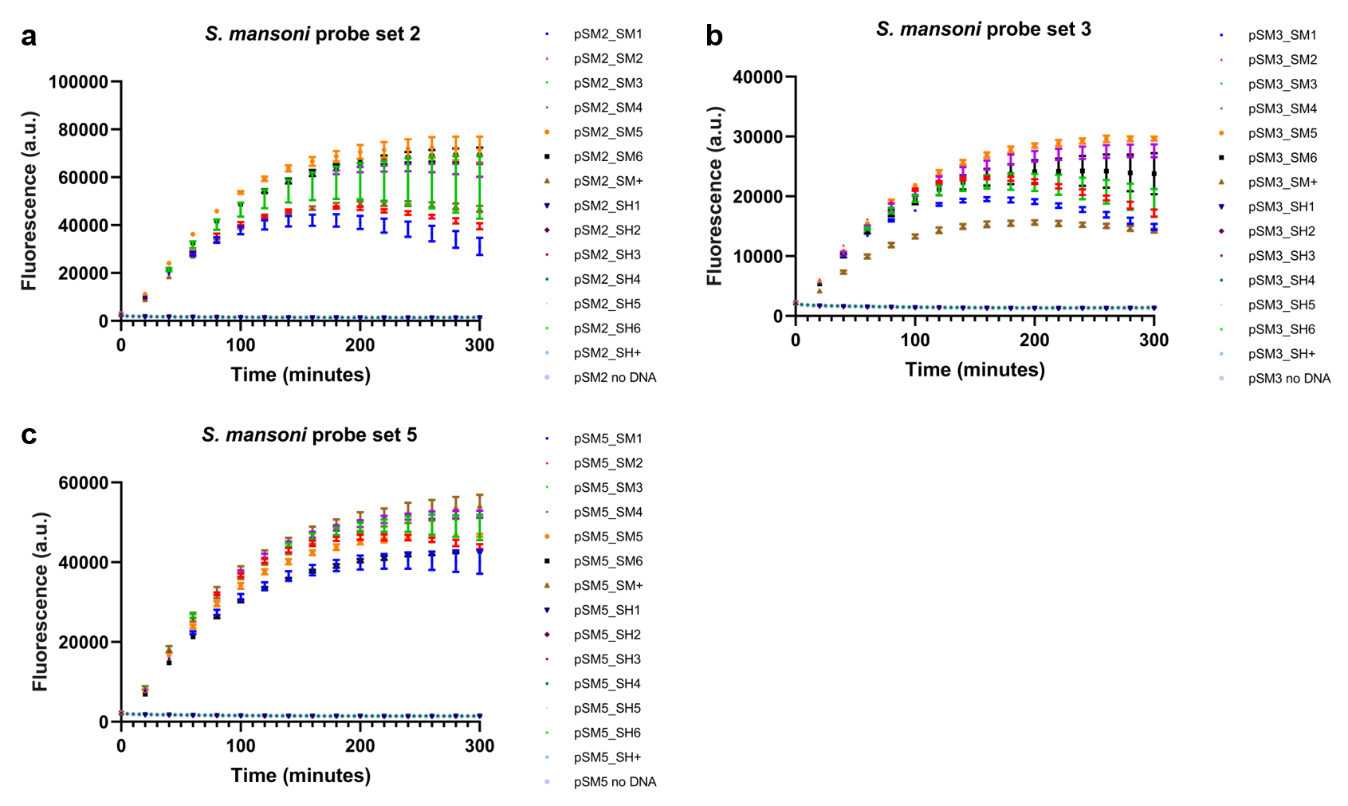
**

**Figure L in S1 Text. Validation of new *S. mansoni*-specific probe sets against ssDNA derived from plasmid DNA.** Graphs represent **a**, probe set 2, **b**, probe set 3 and **c**, probe set 5. 50 nM of each probe half was incubated with 30 ng of the purified ssDNA samples amplified from plasmids pAJW250 (*S. mansoni* insert DNA) and pAJW251 (*S. haematobium* insert DNA) and treated as described in main paper text. Reactions are identified as follows: pSM2_SM1-6, pSM3_SM1-6 and pSM5_SM1-6 (*S. mansoni* probes 2, 3 or 5 against *S. mansoni* plasmid derived ssDNA), pSM2_SM+, pSM3_SM+ and pSM5_SM+ (*S. mansoni* probes 2, 3 or 5 against *S. mansoni* synthetic target), pSM2_SH1-6, pSM3_SH1-6 and pSM5_SH1-6 (*S. mansoni* probes 2, 3 or 5 against *S. haematobium* plasmid derived ssDNA), pSM2_SH+, pSM3_SH+ and pSM5_SH+ (*S. mansoni* probes 2, 3 or 5 against *S. haematobium* synthetic target). *n* = 3 (1 replicate per each reaction split into triplicate runs). Error bars denote standard error of the mean.

**
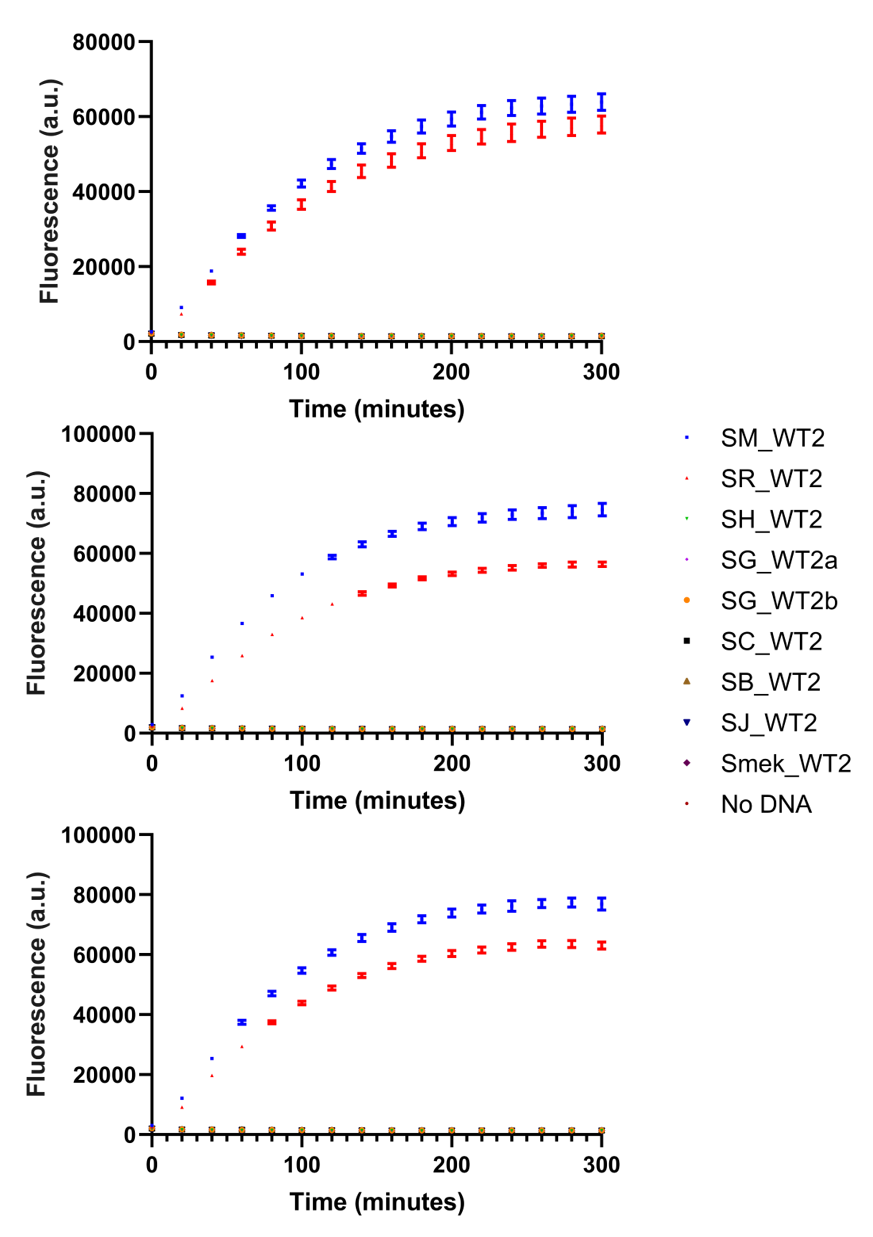
**

**Figure M in S1 Text. Specificity of *S. mansoni* probe 2.** Both half probes and the target DNA concentrations were tested at 50 nM. Targets are listed in the key, and further details of these targets are supplied in Table B in S1 Text. Three reaction set runs are shown separately, with *n*=3 per graph (1 replicate per reaction, each reaction split into triplicate runs). Error bars denote standard error of the mean.

**
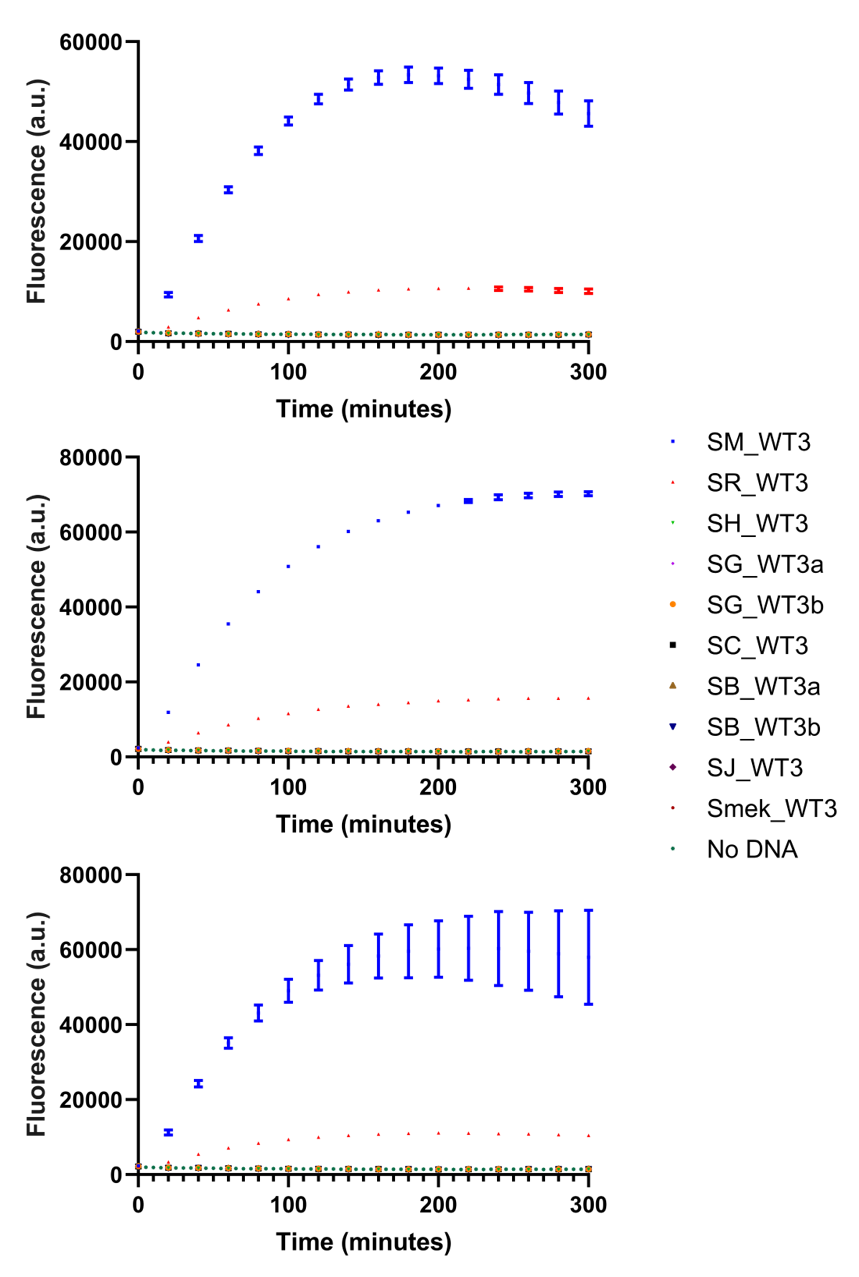
**

**Figure N in S1 Text.** **Specificity of *S. mansoni* probe 3.** Both half probes and the target DNA concentrations were tested at 50 nM. Targets are listed in the key, and further details of these targets are supplied in Table B in S1 Text. Three reaction set runs are shown separately, with *n*=3 per graph (1 replicate per reaction, each reaction split into triplicate runs). Error bars denote standard error of the mean.

**
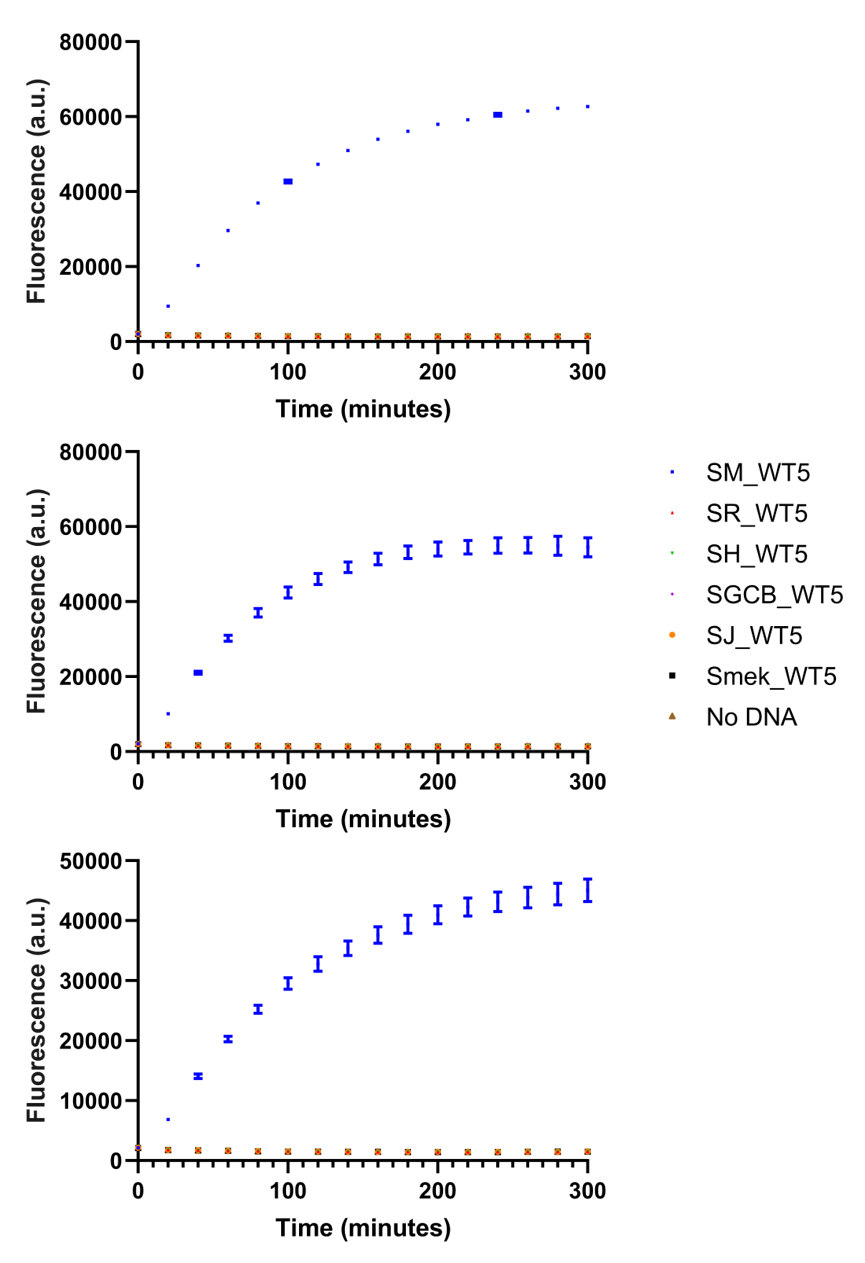
**

**Figure O in S1 Text. Specificity of *S. mansoni* probe 5.** Both half probes and the target DNA concentrations were tested at 50 nM. Targets are listed in the key, and further details of these targets are supplied in Table B in S1 Text. Three reaction set runs are shown separately, with *n*=3 per graph (1 replicate per reaction, each reaction split into triplicate runs). Error bars denote standard error of the mean.


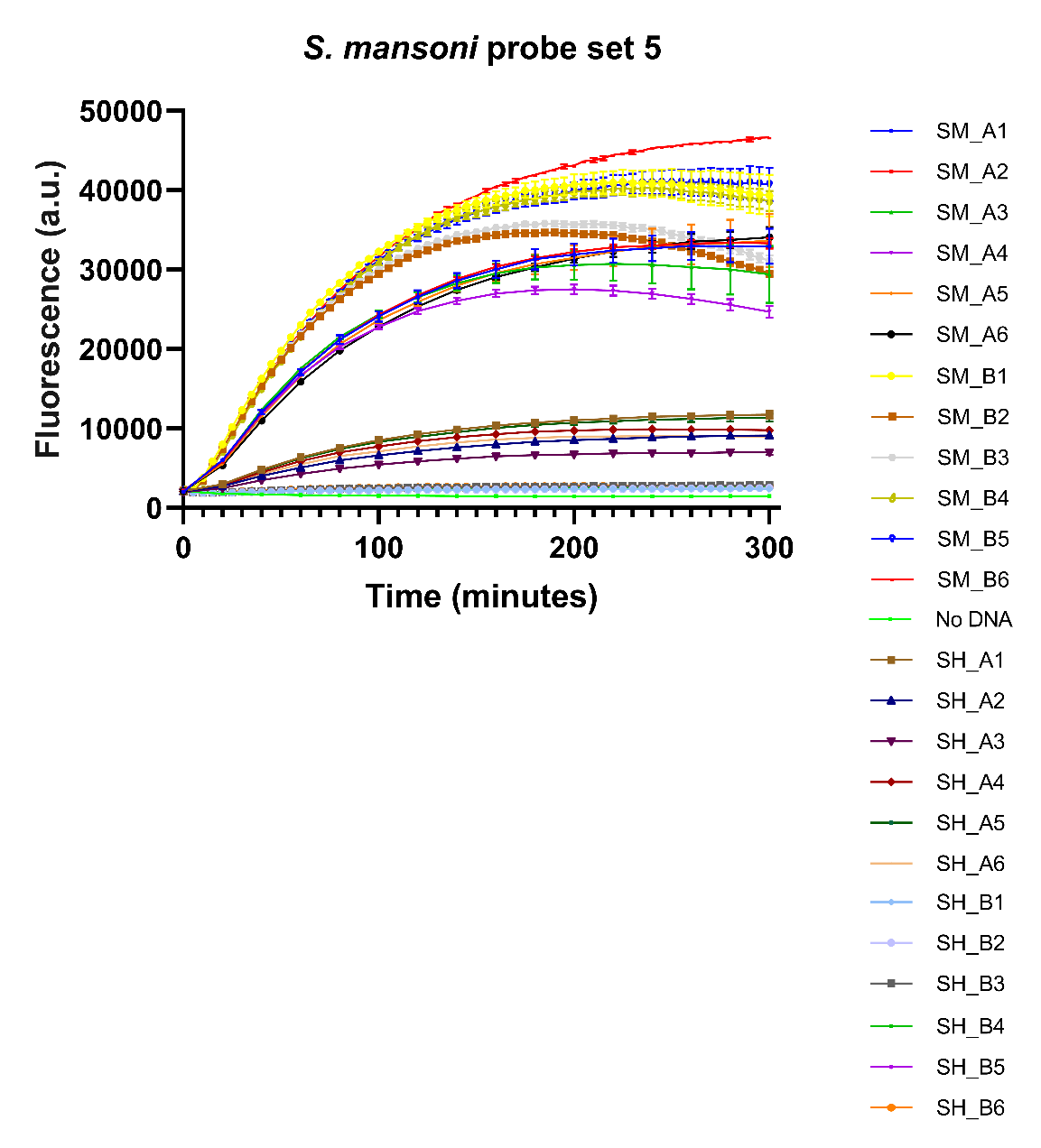


**Figure P in S1 Text. *S. mansoni*-specific probe set 5 can differentiate between *S. mansoni* and *S. haematobium* gDNA-derived ssDNA.** ssDNA derived from adult worm gDNA. 50 nM of each probe half was incubated with 30 ng of purified ssDNA. Reactions are identified as follows: SM_A1-6 (*S. mansoni* adult worm 1 derived ssDNA samples), SM_B1-6 (*S. mansoni* adult worm 2 derived ssDNA samples), SH_A1-6 (*S. haematobium* adult worm 1 derived ssDNA samples), SH_B1-6 (*S. haematobium* adult worm 2 derived ssDNA samples). *n* = 3 (1 replicate per reaction, split into triplicate runs). Error bars denote standard error of the mean.


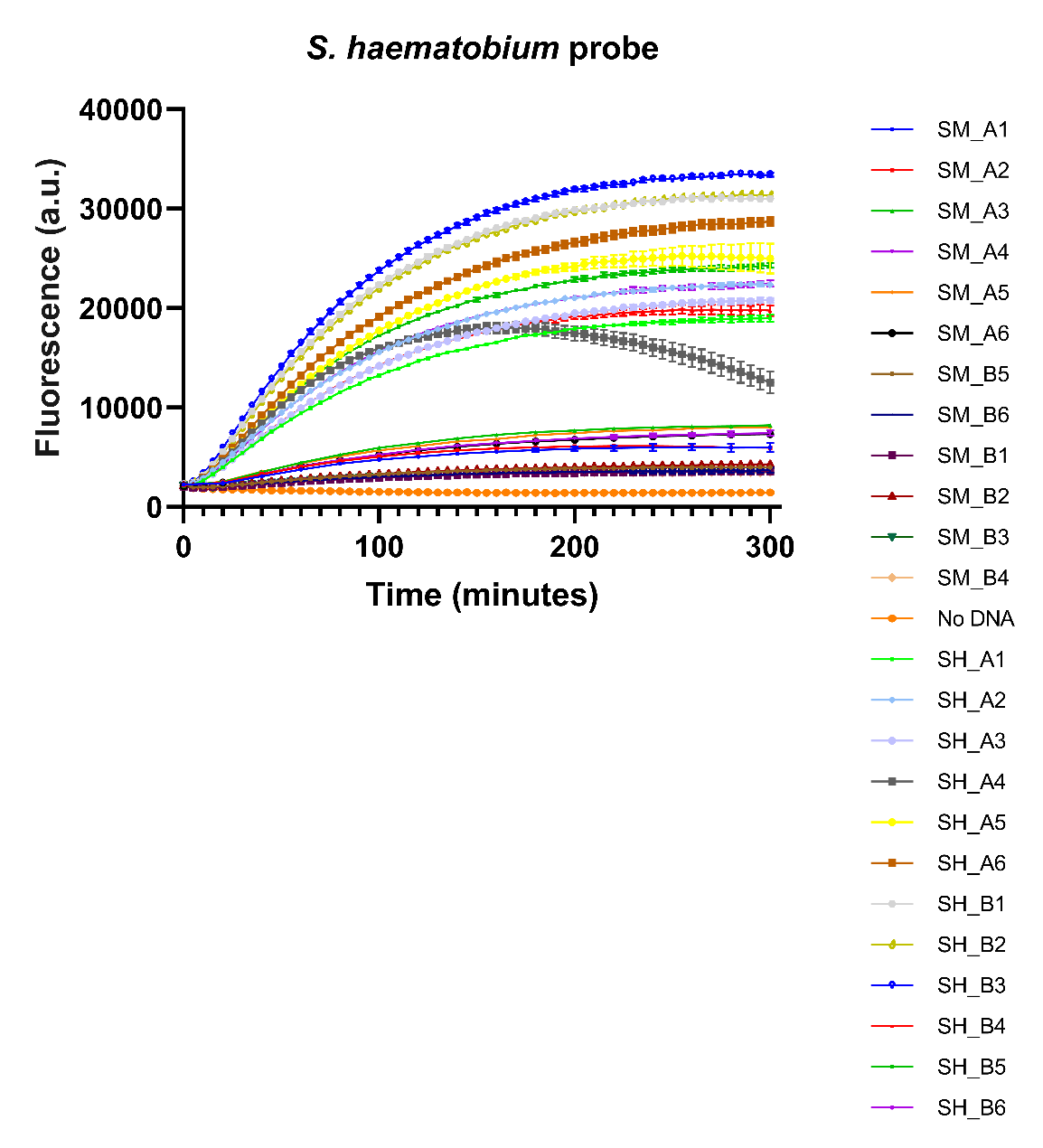


**Figure Q in S1 Text. *S. haematobium*-specific probe set 1 can differentiate between *S. mansoni* and *S. haematobium* gDNA-derived ssDNA.** ssDNA derived from adult worm gDNA. 50 nM of each probe half was incubated with 30 ng of purified ssDNA. Reactions are identified as follows: SM_A1-6 (*S. mansoni* adult worm 1 derived ssDNA samples), SM_B1-6 (*S. mansoni* adult worm 2 derived ssDNA samples), SH_A1-6 (*S. haematobium* adult worm 1 derived ssDNA samples), SH_B1-6 (*S. haematobium* adult worm 2 derived ssDNA samples). *n* = 3 (1 replicate per reaction, split into triplicate runs). Error bars denote standard error of the mean.

**
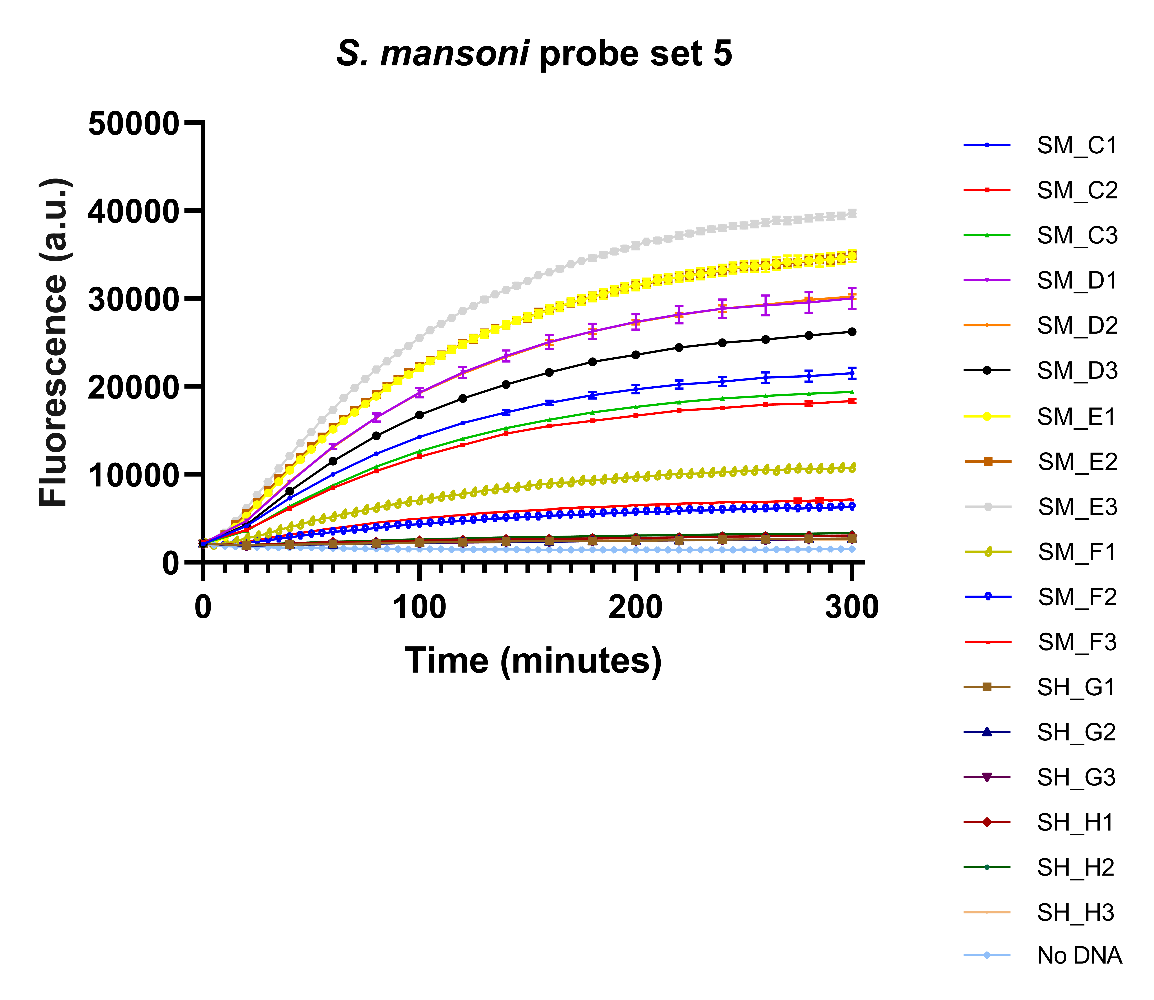
**

**Figure R in S1 Text. Ability of *S. mansoni*-specific probe set 5 to detect *S. mansoni* samples from disparate geographical locations.** ssDNA derived from adult worm gDNA. 50 nM of each probe half was incubated with 30 ng of purified ssDNA. Reactions are identified as follows: SM_C1-3 (*S. mansoni* 2740 derived ssDNA samples), SM_D1-3 (*S. mansoni* 3112 derived ssDNA samples), SM_E1-3 (*S. mansoni* 4209 derived ssDNA samples), SM_F1-3 (*S. mansoni* N1g274 derived ssDNA samples), SM_G1-3 (*S. mansoni* 4464-2 derived ssDNA samples), SM_H1-3 (*S. mansoni* 4464-4 derived ssDNA samples). *n* = 3 (1 replicate per reaction, split into triplicate runs). Error bars denote standard error of the mean.

**
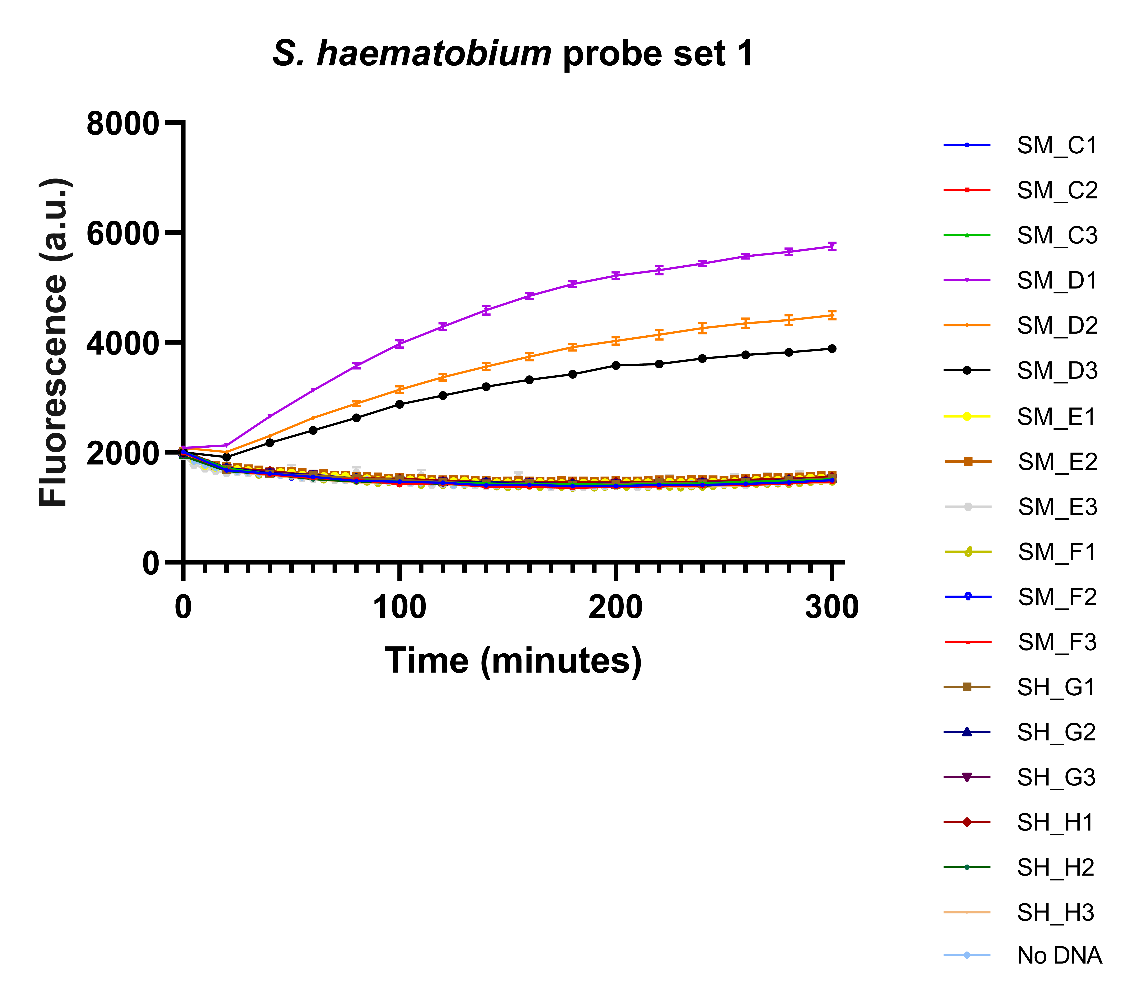
**

**Figure S in S1 Text. Ability of *S. haematobium*-specific probe set 1 to detect *S. mansoni* samples from disparate geographical locations.** ssDNA derived from adult worm gDNA. 50 nM of each probe half was incubated with 30 ng of purified ssDNA. Reactions are identified as follows: SM_C1-3 (*S. mansoni* 2740 derived ssDNA samples), SM_D1-3 (*S. mansoni* 3112 derived ssDNA samples), SM_E1-3 (*S. mansoni* 4209 derived ssDNA samples), SM_F1-3 (*S. mansoni* N1g274 derived ssDNA samples), SM_G1-3 (*S. mansoni* 4464-2 derived ssDNA samples), SM_H1-3 (*S. mansoni* 4464-4 derived ssDNA samples). *n* = 3 (1 replicate per reaction, split into triplicate runs). Error bars denote standard error of the mean.

**Table A in S1 Text. Oligonucleotide probes and primers used in this study**

| **Number or reference** | **Name** | **Sequence (5’-3’)** |
| --- | --- | --- |
| **Probes** | | |
| AJW694 | 2^nd^ T7 promoter sequence | **TAATACGACTCACTATAGGG** |
| AJW789 | SM_A1 | **p-TTAAACGACTAGCGATACCCTATAGTGAGTCGTATTA** |
| AJW790 | SM_B1 | **GTGTGGGAGCCCACACTCTACTCGACAGATACGAATATCTGGACCCGACCGTCTCCCACACACATTTGAATC** |
| AJW848 | SH_A1 | **p-TGACACGACTAGCGATACCCTATAGTGAGTCGTATTA** |
| AJW849 | SH_B1 | **GTGTGGGAGCCCACACTCTACTCGACAGATACGAATATCTGGACCCGACCGTCTCCCACACAGTCTTAAAAT** |
| AJW884 | SM_A2 | **p-ACTTGAAACCCGCGATACCCTATAGTGAGTCGTATTA** |
| AJW885 | SM_B2 | **GTGTGGGAGCCCACACTCTACTCGACAGATACGAATATCTGGACCCGACCGTCTCCCACACGATCCAATTAG** |
| AJW886 | SM_A3 | **p-CCAATTAGACTGCGATACCCTATAGTGAGTCGTATTA** |
| AJW887 | SM_B3 | **GTGTGGGAGCCCACACTCTACTCGACAGATACGAATATCTGGACCCGACCGTCTCCCACACAATTGACAGAT** |
| AJW890 | SM_A5 | **p-GTAAATAGATAGCGATACCCTATAGTGAGTCGTATTA** |
| AJW891 | SM_B5 | **GTGTGGGAGCCCACACTCTACTCGACAGATACGAATATCTGGACCCGACCGTCTCCCACACGTAAGACAGAC** |
| **Targets** | | |
| AJW791 | SM_WT | TAGTCGTTTAAGATTCAAATGT |
| AJW792 | SM_B | TAGTCGTTTAACATTCAAATGT |
| AJW793 | SM_C | TAGTCGTTTAAAATTCAAATGT |
| AJW794 | SM_D | TAGTCGTTTAATATTCAAATGT |
| AJW795 | SM_E | TAGTCGTTTATGATTCAAATGT |
| AJW796 | SM_F | TAGTCGTTTAGGATTCAAATGT |
| AJW797 | SM_G | TAGTCGTTTACGATTCAAATGT |
| AJW798 | SM_H | TAGTCGTTTATCATTCAAATGT |
| AJW799 | SM_I | TAGTCGTTTATAATTCAAATGT |
| AJW800 | SM_J | TAGTCGTTTATTATTCAAATGT |
| AJW801 | SM_K | TAGTCGTTTAGCATTCAAATGT |
| AJW802 | SM_L | TAGTCGTTTAGAATTCAAATGT |
| AJW803 | SM_M | TAGTCGTTTAGTATTCAAATGT |
| AJW804 | SM_N | TAGTCGTTTACCATTCAAATGT |
| AJW805 | SM_O | TAGTCGTTTACAATTCAAATGT |
| AJW806 | SM_P | TAGTCGTTTACTATTCAAATGT |
| AJW807 | SM_Q | TAGTCGCTTACTATTCAAATGT |
| AJW808 | SM_R | TAGTCGTTTACTATTAAAATGT |
| AJW809 | SM_S | TAGCCGTGTAAGATGCAACTGT |
| AJW810 | SH_WT | TAGTCGTGTCAATTTTAAGACT |
| AJW840 | SR_WT | AAGTCGTTTAAGGTTGAAGTGT |
| AJW841 | SB_WT | GAGTTGTGTAGATTTTAAGACT |
| AJW842 | SG_WT | TAGTTGTGTTGATTTTAAGACT |
| AJW843 | SC_WT | CAGTCGTGTTGATTTTAAGACT |
| AJW844 | SJ_WT | GTTGCGTCTAAGGTCATGTTCT |
| AJW845 | Smek_WT | GATACGTTTAAATTCTTGTTCT |
| AJW874 | SM_180 | CTGGTTTTGGTGTAGATTATTTAATGTTTTCTCTTCATTTGGCAGGGGTTTCAAGTCTAATTGGATCTGTCAATTTCATTTCTACGATTTTTAGTCGTTTAAGATTCAAATGTTCGATAATAGTATGGGCTTATCTATTTACGTCTGTCTTACTATTGCTTTCATTACCTGTATTAGCCA |
| AJW875 | SH_180 | CGGGTTTAGGTGTAGATTATTTAATGTTTTCTTTACATTTAGCGGGTGTATCTAGATTAGTTGGTTCCATTAATTTTATTTCTACGATTATTAGTCGTGTCAATTTTAAGACTTCTATAATAATATGATCATATTTGTTCACTTCTATCTTATTATTGCTTTCATTACCAGTTTTAGCAG |
| AJW894 | SM_WT_2 | GGGTTTCAAGTCTAATTGGATC |
| AJW895 | SR_WT_2 | GAGTTTCAAGTTTAATTGGTTC |
| AJW896 | SH_WT_2 | GTGTATCTAGATTAGTTGGTTC |
| AJW897 | SG_WT_2 | GTGTGTCTAGGTTGGTTGGTTC |
| AJW898 | SG_WT_2_AJ519522 | GTGTGTCTAGGTTGGTTGGCTC |
| AJW899 | SC_WT_2 | GTGTGTCTAGGTTAGTTGGTTC |
| AJW900 | SB_WT_2 | GTGTGTCTAGATTGGTTGGTTC |
| AJW901 | SJ_WT_2 | GTGTATCTAGTTTGATTGGTTC |
| AJW902 | Smek_WT_2 | GTATTTCTAGTTTGATAGGTTC |
| AJW903 | SM_WT_3 | AGTCTAATTGGATCTGTCAATT |
| AJW904 | SR_WT_3 | AGTTTAATTGGTTCTGTTAATT |
| AJW905 | SH_WT_3 | AGATTAGTTGGTTCCATTAATT |
| AJW906 | SG_WT_3 | AGTTTGGTTGGTTCAATTAATT |
| AJW907 | SG_WT_3_AJ519522 | AGTTTGGTTGGCTCAATTAATT |
| AJW908 | SC_WT_3 | AGGTTAGTTGGTTCAATTAATT |
| AJW909 | SB_WT_3 | AGATTGGTTGGTTCAATTAATT |
| AJW910 | SB_WT_3_FJ897160 | AGATTGGTTGGTTCAATCAATT |
| AJW911 | SJ_WT_3 | AGTTTGATTGGTTCTATAAATT |
| AJW912 | Smek_WT_3 | AGTTTGATAGGTTCTGTGAAAT |
| AJW913 | SM_WT_5 | TATCTATTTACGTCTGTCTTAC |
| AJW914 | SR_WT_5 | TATTTATTTACATCTATTCTTT |
| AJW915 | SH_WT_5 | TATTTGTTCACTTCTATCTTAT |
| AJW916 | SG_SC_SB_WT_5 | TATTTATTTACTTCAATTTTAT |
| AJW917 | SJ_WT_5 | TATTTATTTACTTCGGTGTTGT |
| AJW918 | Smek_WT_5 | TATTTTTTTACTTCTATACTTT |
| **Primers for target amplification** | | |
| AJW485 | Cox1_schist_5’ | TCTTTRGATCATAAGCG |
| AJW486 | Cox1_schist_3’ | TAATGCATMGGAAAAAAACA |
| AJW870 | 5-SM-PCR | CTGGTTTTGGTGTAGATTATTTAATG |
| AJW871 | 3-SM-PCR | p-TGGCTAATACAGGTAATGAAAGCAAT |
| AJW872 | 5-SH-PCR | CGGGTTTAGGTGTAGATTATTTAATG |
| AJW873 | 3-SH-PCR | p-CTGCTAAAACTGGTAATGAAAGCAAT |
| **Primers for sequencing** | | |
| AJW8 | M13 Rev | CAGGAAACAGCTATGAC |
| AJW9 | M13 Fwd | GTAAAACGACGGCCAG |
| AJW487 | 5-Seq-SHcox1-A | GCTTAAAAGCTGTGGGTCTCGTG |
| AJW488 | 3-Seq-SHcox1-A | CCAAATCCACCTATCAATATAGGC |
| AJW489 | 5-Seq-SMcox1-A | GGTGTCACAGGGGTGGCTTTATCTGC |
| AJW490 | 3-Seq-SMcox1-A | GCAGATAAAGCCACCCCTGTGACACC |

For the probes, the target sequences, T7 promoter sequence and the ‘Spinach’ aptamer sequence are indicated by blue, red and green text respectively. Phosphorylated primers are indicated by ‘p-’. The sequence of the 2^nd^ T7 promoter sequence primer (AJW694) was taken from[2], and the sequences of primers Cox1_schist_5’ (AJW485) and Cox1_schist_3’ (AJW486) were taken from[3].

**Table B in S1 Text. GenBank accession numbers of the *cox*1 gene sequences used to identify biosensor targets**

| **Species** | **Accession number** | **Reference** |
| --- | --- | --- |
| *S. mansoni* | AJ519524 | [4] |
| *S. mansoni* | MG562513 | GenBank |
| *S. rodhaini* | AY157202 | [3] |
| *S. haematobium* | GU257338 | [5] |
| *S. haematobium* | GU257336 | [5] |
| *S. bovis* | MH647124 | [6] |
| *S. bovis* | FJ897160 | [7] |
| *S. guineensis* | AJ519517 | [4] |
| *S. guineensis* | AJ519522 | [4] |
| *S. guineensis* | AJ519523 | [4] |
| *S. curassoni* | AJ519516 | [4] |
| *S. curassoni* | AY157210 | [3] |
| *S. japonicum* | EU325878 | GenBank |
| *S. malayensis* | EF635956 | [8] |
| *S. mekongi* | EF635955 | [8] |

**Table C in S1 Text. Bacterial strains and constructs used in this study.**

| **Strain or plasmid** | **Relevant features** | **Reference(s)** |
| --- | --- | --- |
| **Strain** |  |  |
| NEB10-beta | Δ(*ara* -*leu*) 7697 *araD139 fhuA* Δ*lacX74* *galK16 galE15 e14*- ϕ*80*d*lacZ*Δ*M15* *recA1 relA1endA1 nupG rpsL* (StrR) *rph spoT*1 Δ(*mrrhsdRMS-mcrBC*); Cloning strain | New England Biolabs |
| NEB5-alpha | *fhuA2* Δ(*argF-lacZ*) *U169 phoA glnV44* ϕ*80*Δ(*lacZ*) *M15 gyrA96 recA1 relA1 endA1 thi-1 hsdR17*: Cloning strain | New England Biolabs |
| **Plasmid** |  |  |
| pCR-Blunt | Cloning vector for PCR products; KanR, ZeoR | Invitrogen |
| pCR-Blunt II-TOPO | Cloning vector for PCR products; KanR, ZeoR | Invitrogen |
| pAJW250 | NEB10-beta pCR-Blunt-SM-*cox*1; *S. mansoni* *cox*1 gene fragment amplified using primers Cox1_schist_5’ and Cox1_schist_3’; Amplified from *S. mansoni* adult worm 1 gDNA; KanR, ZeoR | This study |
| pAJW251 | NEB10-beta pCR-Blunt-SH-*cox*1; *S. haematobium* *cox*1 gene fragment amplified using primers Cox1_schist_5’ and Cox1_schist_3’; Amplified from *S. haematobium* adult worm 1 gDNA; KanR, ZeoR | This study |
| pAJW314 | NEB10-beta pCR-Blunt-SM-*cox*1-180bp-SMAW1 clone 1; *S. mansoni* *cox*1 180 base pair gene fragment amplified using primers 5-SM-PCR and 3-SM-PCR; Amplified from *S. mansoni* adult worm 1 gDNA; KanR, ZeoR | This study |
| pAJW315 | NEB10-beta pCR-Blunt-SM-*cox*1-180bp-SMAW1 clone 2; *S. mansoni* *cox*1 180 base pair gene fragment amplified using primers 5-SM-PCR and 3-SM-PCR; Amplified from *S. mansoni* adult worm 1 gDNA; KanR, ZeoR | This study |
| pAJW316 | NEB10-beta pCR-Blunt-SM-*cox*1-180bp-SMAW2clone 1; *S. mansoni* *cox*1 180 base pair gene fragment amplified using primers 5-SM-PCR and 3-SM-PCR; Amplified from *S. mansoni* adult worm 2 gDNA; KanR, ZeoR | This study |
| pAJW317 | NEB10-beta pCR-Blunt-SM-*cox*1-180bp-SMAW2 clone 2; *S. mansoni* *cox*1 180 base pair gene fragment amplified using primers 5-SM-PCR and 3-SM-PCR; Amplified from *S. mansoni* adult worm 2 gDNA; KanR, ZeoR | This study |
| pAJW318 | NEB10-beta pCR-Blunt-SH-*cox*1-180bp-SHAW1 clone 1; *S. haematobium* *cox*1 180 base pair gene fragment amplified using primers 5-SH-PCR and 3-SH-PCR; Amplified from *S. haematobium* adult worm 1 gDNA; KanR, ZeoR | This study |
| pAJW319 | NEB10-beta pCR-Blunt-SH-*cox*1-180bp-SHAW1 clone 2; *S. haematobium* *cox*1 180 base pair gene fragment amplified using primers 5-SH-PCR and 3-SH-PCR; Amplified from *S. haematobium* adult worm 1 gDNA; KanR, ZeoR | This study |
| pAJW320 | NEB10-beta pCR-Blunt-SH-*cox*1-180bp-SHAW2 clone 1; *S. haematobium* *cox*1 180 base pair gene fragment amplified using primers 5-SH-PCR and 3-SH-PCR; Amplified from *S. haematobium* adult worm 2 gDNA; KanR, ZeoR | This study |
| pAJW321 | NEB10-beta pCR-Blunt-SH-*cox*1-180bp-SHAW2 clone 2; *S. haematobium* *cox*1 180 base pair gene fragment amplified using primers 5-SH-PCR and 3-SH-PCR; Amplified from *S. haematobium* adult worm 2 gDNA; KanR, ZeoR | This study |
| pAJW323 | NEB5-alpha pCR-Blunt II-TOPO-SM-*cox*1; *S. mansoni* *cox*1 gene fragment amplified using primers Cox1_schist_5’ and Cox1_schist_3’; Amplified from *S. mansoni* 2740 gDNA; KanR, ZeoR | This study |
| pAJW324 | NEB5-alpha pCR-Blunt II-TOPO-SM-*cox*1; *S. mansoni* *cox*1 gene fragment amplified using primers Cox1_schist_5’ and Cox1_schist_3’; Amplified from *S. mansoni* 3112 gDNA; KanR, ZeoR | This study |
| pAJW325 | NEB5-alpha pCR-Blunt II-TOPO-SM-*cox*1; *S. mansoni* *cox*1 gene fragment amplified using primers Cox1_schist_5’ and Cox1_schist_3’; Amplified from *S. mansoni* 4209 gDNA; KanR, ZeoR | This study |
| pAJW326 | NEB5-alpha pCR-Blunt II-TOPO-SM-*cox*1; *S. mansoni* *cox*1 gene fragment amplified using primers Cox1_schist_5’ and Cox1_schist_3’; Amplified from *S. mansoni* N1g274 gDNA; KanR, ZeoR | This study |
| pAJW327 | NEB5-alpha pCR-Blunt II-TOPO-SM-*cox*1; *S. mansoni* *cox*1 gene fragment amplified using primers Cox1_schist_5’ and Cox1_schist_3’; Amplified from *S. mansoni* 4464-2 gDNA; KanR, ZeoR | This study |
| pAJW328 | NEB5-alpha pCR-Blunt II-TOPO-SM-*cox*1; *S. mansoni* *cox*1 gene fragment amplified using primers Cox1_schist_5’ and Cox1_schist_3’; Amplified from *S. mansoni* 4464-4 gDNA; KanR, ZeoR | This study |

**Table D in S1 Text. Sequences of *cox*1 amplified from *S. mansoni* and *S. haematobium* adult worm gDNA and cloned into pCR-Blunt**

| **Plasmid construct** | **Notes** | **Sequence** |
| --- | --- | --- |
| pAJW250 | *cox*1 amplified from *S. mansoni* adult gDNA (*S. mansoni* adult worm 1) using primers AJW485 and AJW486 and cloned into pCR-Blunt. Sequence verified using plasmid specific primers AJW8/AJW9 and insert specific primers AJW489/AJW490. | TCTTTRGATCATAAGCGTATAGGGTTATTATATTTTGTTTTTGGGTTATGAGGTGGGTTTATTGGTTTAGGTTTAAGGTTATTAATTCGTTTAAATTTATGTGATCCTTATTATAAATTGGTTTCTGTAGATGTTTATAATTATTTGGTTACTAATCATGGTGTAGCTATGATTTTTTTTTTTTTGATGCCAATTTTAATAGGTGGATTTGGTAATTATTTCCTTCCAATATTTTTATGTTTAGATGATTTGTTATTGCCTCGTTTAAATTCTTTAAGATTATGATTGATGGTTCCTTCTATTTTTTATATGGAACTTAGGTTATATTATGGGTGTGGAATTGGTTGAACTCTATATCCTCCTTTATCAATTTGAGAGGGGTCTGGTTTTGGTGTAGATTATTTAATGTTTTCTCTTCATTTGGCAGGGGTTTCAAGTCTAATTGGATCTGTCAATTTCATTTCTACGATTTTTAGTCGTTTAAGATTCAAATGTTCGATAATAGTATGGGCTTATCTATTTACGTCTGTTTTATTATTGCTTTCGTTACCTGTGTTAGCCAGAGGAATAACGATGTTATTATTTGATCGTAAATTTGGAACTGCTTTTTTTGAGCCGTCAGGCGGTGGCGATCCTATTTTGTTTCAGCATTTATTTTGGTTTTTTGGTCATCCAGAGGTTTATGTTTTGATCCTTCCGGGTTTTGGTATAGTTAGGCATATCTGTATGAGTCTAAGGAATAAAGATTCGTCGTTTGGTTATTATGGATTGATTTGCGCTATGGCTTCCATAGTATGCTTAGGTAGAGTAGTATGGGGTCATCATATGTTTATGGTTGGCTTTGATTCGTTAACTGGAGTGTTTTTTAGTTCTATTACTATGATAATAGGTGTTCCTACTGGTATTAAGGTGTTTTCATGACTTTATATGTTGAATAGTTGTGGTATGCGGGTTTTAGATCCCATAGTATGGTGATTAGTCGGTTTTATATTTTTATTTACGGTTGGTGGTGTCACAGGGGTGGCTTTATCTGCATCTGCTTTAGATATACTGTTTCATGATACTTGGTTTGTTGTTGCTCATTTTCATTACGTTCTTTCTTTAGGTTCTTATAGAAGAATAGTGATAATGCTAGTATGGTGATGGCCGTTTATAGTTGGTTATAGTTTAAATAAGTATTTATTACAAGGTCACTGATTATTATCTATGGTTGGTTTTAACTTGTGTTTTTTTCCTATGCATTA |
| pAJW251 | *cox*1 amplified from *S. haematobium* adult gDNA (*S. haematobium* adult worm 1) using primers AJW485 and AJW486 and cloned into pCR-Blunt. Sequence verified using plasmid specific primers AJW8/AJW9 and insert specific primers AJW487/AJW488. | TCTTTRGATCATAAGCGGGGGGTTTTATTGGTTTAGGTTTGAGGCTTTTAATTCGATTAAATTTATGTGATCCATATTATAATTTGGTTTCATTAGATGTTTATAAATTTTTGATTACTAACCATGGTATAGCTATGATTTTTTTTTTTTTAATGCCTATATTGATAGGTGGATTTGGTAAATATTTTCTTCCGTTTTTTTTATATATAGATGATTTGTTGTTACCTCGATTGAATTCTTTTAGTTTATGATTAATGATTCCTTCATTTTTTTATATGGAGTTGAGTTTATACTATGGTTGTGGTGTAGGATGAACATTGTATCCTCCATTATCCATATCTGAGAATTCAGGTTTAGGTGTAGATTATTTAATGTTTTCTTTACATTTAGCGGGTGTATCTAGATTAGTTGGTTCTATTAATTTTATTTCTACGATTATTAGTCGTGTCAATTTTAAGACTTCTATAATAATATGGTCGTATTTGTTTACTTCTATTTTATTATTGCTTTCATTGCCAGTTTTAGCAGCTGGTATTACTATGCTATTATTTGATCGTAAATTTGGTACTGCTTTTTTTGAGCCTATGGGTGGTGGTGATCCATTATTATTTCAGCACTTATTTTGATTTTTTGGTCATCCAGAGGTGTATGTTTTAATTTTACCTGGATTTGGAATAGTTAGTCATATATGTATGAGGATAAGTAATAATGATTCATCGTTTGGGTATTATGGATTGATTTGTGCTATGGCTTCGATAGTTTGCTTAGGAAGTGTAGTTTGAGCCCATCATATGTTTATGGTTGGTTTAGATTATTTGACTGCTATATTTTTTAGTTCAGTGACTATGATTATAGGGATTCCTACAGGTATAAAGGTTTTTTCTTGATTATATATGCTTAAAAGCTGTGGGTCTCGTGTATGAGATCCTATAGTTTGATGATTGGTTGGTTTTATATTTTTATTTACGATAGGTGGTGTTACTGGTATAGCTTTATCAGCTTCTTCATTAGATATATTATTTCATGATACTTGATTTGTTGTTGCTCATTTTCATTATGTTCTTTCTTTAGGCTCTTATAGAAGTGTAGTAATAATGTTATTATGGTGATGGCCTTTTATAATTGGTTACAGTATTAATAAGTAATGCATMGGAAAAAAACA |

Only *cox*1 insert sequences are shown in the table.

**Table E in S1 Text. Sequences of *cox*1 180-base targets PCR amplified from *S. mansoni* and *S. haematobium* adult worm gDNA and cloned into pCR-Blunt or pCR-Blunt II-TOPO**

| **Plasmid construct** | **Notes** | **Sequence** |
| --- | --- | --- |
| pAJW314/pAJW315 | *cox*1 180 base pair gene fragment amplified from *S. mansoni* adult worm 1 gDNA using primers 5-SM-PCR and 3-SM-PCR. | CTGGTTTTGGTGTAGATTATTTAATGTTTTCTCTTCATTTGGCGGGGGTTTCAAGTCTAATTGGATCTGTCAATTTCATTTCTACGATTTTTAGTCGTTTAAGATTCAAATGTTCGATAATAGTATGGGCT**TATCTATTTACGTCTGTCTTAC**TATTGCTTTCATTACCTGTATTAGCCA |
| pAJW316/pAJW317 | *cox*1 180 base pair gene fragment amplified from *S. mansoni* adult worm 2 gDNA using primers 5-SM-PCR and 3-SM-PCR. | CTGGTTTTGGTGTAGATTATTTAATGTTTTCTCTTCATTTGGCAGGGGTTTCAAGTCTAATTGGATCTGTCAATTTCATTTCTACGATTTTTAGTCGTTTAAGATTCAAATGTTCGATAATAGTATGGGCT**TATCTATTTACGTCTGTCTTAC**TATTGCTTTCATTACCTGTATTAGCCA |
| pAJW318/pAJW319 | *cox*1 180 base pair gene fragment amplified from *S. haematobium* adult worm 1 gDNA using primers 5-SH-PCR and 3-SH-PCR. | CGGGTTTAGGTGTAGATTATTTAATGTTTTCTTTACATTTAGCGGGTGTATCTAGATTAGTTGGTTCTATTAATTTTATTTCTACGATTAT**TAGTCGTGTCAATTTTAAGACT**TCTATAATAATATGGTCGTATTTGTTTACTTCTATTTTATTATTGCTTTCATTACCAGTTTTAGCAG |
| pAJW320/pAJW321 | *cox*1 180 base pair gene fragment amplified from *S. haematobium* adult worm 2 gDNA using primers 5-SH-PCR and 3-SH-PCR. | CGGGTTTAGGTGTAGATTATTTAATGTTTTCTTTACATTTAGCGGGTGTATCTAGATTAGTTGGTTCCATTAATTTTATTTCTACGATTAT**TAGTCGTGTCAATTTTAAGACT**TCTATAATAATATGATCATATTTGTTCACTTCTATCTTATTATTGCTTTCATTACCAGTTTTAGCAG |
| pAJW323 | sequence of the *cox*1 180 base pair gene fragment from *S. mansoni* 2740. Target sequence subset 5. | CTGGTTTTGGTGTAGATTATTTAATGTTTTCTCTTCATTTGGCAGGGGTTTCAAGTCTAATTGGATCTGTCAATTTCATTTCTACGATTTTTAGTCGTTTAAGATTCAAATGTTCGATAATAGTATGGGCT**TATCTATTTACGTCTGTTTTAT**TATTGCTTTCGTTACCTGTGTTAGCCA |
| pAJW324 | sequence of the *cox*1 180 base pair gene fragment from *S. mansoni* 3112. Target sequence subset 3. | CTGGTTTTGGTGTAGATTATTTAATGTTTTCTCTTCATTTGGCAGGGGTTTCAAGTCTAATTGGATCTGTCAATTTCATTTCTACGATTTTTAGTCGTTTAAGATTCAAATGTTCGATAATAGTATGGGCT**TATCTATTTACGTCTATCTTAC**TATTGCTTTCATTACCTGTATTAGCCA |
| pAJW325 | sequence of the *cox*1 180 base pair gene fragment from *S. mansoni* 4209. Wildtype target sequence. | CTGGTTTTGGTGTAGATTATTTAATGTTTTCTCTTCATTTGGCAGGGGTTTCAAGTCTAATTGGATCTGTCAATTTCATTTCTACGATTTTTAGTCGTTTAAGATTCAAATGTTCAATAATAGTATGGGCT**TATCTATTTACGTCTGTCTTAC**TATTGCTTTCATTACCTGTGTTAGCCA |
| pAJW326 | sequence of the *cox*1 180 base pair gene fragment from *S. mansoni* N1g274. Target sequence subset 2. | CTGGTTTTGGTGTAGATTATTTAATGTTTTCTCTTCATTTGGCAGGGGTTTCAAGTCTAATTGGATCTGTTAATTTTATTTCTACGATTTTTAGTCGTTTAAGATTAAAGTGTTCGATAATAGTATGGGCT**TATTTATTTACGTCTGTTTTAC**TGTTGCTTTCATTACCTGTGTTAGCCA |
| pAJW327 | sequence of the *cox*1 180 base pair gene fragment from *S. mansoni* 4464-2. Target sequence subset 2. | CTGGTTTTGGTGTAGATTATTTAATGTTTTCTCTTCATTTGGCAGGGGTTTCAAGTCTAATTGGATCTGTTAATTTTATTTCTACGATTTTTAGTCGTTTAAGATTAAAGTGTTCGATAATAGTATGGGCT**TATTTATTTACGTCTGTTTTAC**TATTGCTTTCATTACCTGTGTTAGCCA |
| pAJW328 | sequence of the *cox*1 180 base pair gene fragment from *S. mansoni* 4464-4. Target sequence subset 2. | CTGGTTTTGGTGTAGATTATTTAATGTTTTCTCTTCATTTGGCAGGGGTTTCAAGTCTAATTGGATCTGTTAATTTTATTTCTACGATTTTTAGTCGTTTAAGATTAAAGTGTTCGATAATAGTATGGGCT**TATTTATTTACGTCTGTTTTAC**TATTGCTTTCATTACCTGTGTTAGCCA |

Only *cox*1 insert sequences are shown in the table. Sequence changes to that expected from alignments shown in Figure K in S1 Text are highlighted in red. The relevant target region for *S. mansoni* probe set 5 and *S. haematobium* probe set 1 are underlined and emboldened on the respective sequence.

**Table F in S1 Text. Sequences used for natural sequence variation analysis of the *cox*1 target for *S. mansoni*-specific probe set 5**

| **Subset** | **Accession number** | **Isolate** | **Geographical**  **location** | **ref** |
| --- | --- | --- | --- | --- |
| SM_WT | MG562513  AJ519524  MN593380  MK172833  MK172834  MK172830  MN593376  MF919421  MN593432  MF919422  MN593388  MF919417  MN593424  MF919427  MN593384  MF919418  MF919416 | Sm_Coi5  2797  E56_hsDJ  NCRr  NCBp  DC1Hs  E55_hsDJ  X150  Y205_mhGU  X228  E59_hsTE  X71  Y15_mhMG  X2  E58_hsND  X234  X70 | Cote d'Ivoire  Senegal  Senegal: Didjiery  Oman: Dhofar Tibraq  Oman: Dhofar Tibraq  Oman: Dhofar Tibraq  Senegal: Didjiery  Senegal: Temey, Lac de Guiers  Senegal: Gueo  Senegal: Temey, Lac de Guiers  Senegal: Temey  Senegal: Richard Toll  Senegal: Merina Guewel  Senegal: Nder, Lac de Guiers  Senegal: Nder  Senegal: Temey, Lac de Guiers  Senegal: Richard Toll | Genbank  [4]  [9]  [10]  [10]  [10]  [9]  [11]  [9]  [11]  [9]  [11]  [9]  [11]  [9]  [11]  [11] |
| Subset 1 | MF919425 | X260 | Senegal: Temey, Lac de Guiers | [11] |
| Subset 2 | MN593408  MF919423 | X72_anNG  X72 | Senegal: Richard Toll  Senegal: Richard Toll | [9]  [11] |
| Subset 3 | MF919426  MN593404  MF919419  MF919428 | X185  E70_bpND  X235  X153 | Senegal: Temey, Lac de Guiers  Senegal: Nder  Senegal: Temey, Lac de Guiers  Senegal: Temey, Lac de Guiers | [11]  [9]  [11]  [11] |
| Subset 4 | MT994261  MK172832  MK172831  MG562512  MF919424 | S.mans_H1  DC3Hs  DC2Hs  Sm_Coi1  X139 | Zimbabwe  Oman: Dhofar Sheer  Oman: Dhofar Sheer  Cote d'Ivoire  Senegal: Temey, Lac de Guiers | [12]  [10]  [10]  Genbank  [11] |
| Subset 5 | KX011043 |  | Gabon | [13] |
| Subset 6 | MF919420 | X145 | Senegal: Temey, Lac de Guiers | [11] |

**Table G in S1 Text. Sequences used for natural sequence variation analysis of the *cox*1 target for *S. haematobium*-specific probe set 1**

| **Subset** | **Accession number** | **Haplotype** | **Geographical**  **location** | **ref** |
| --- | --- | --- | --- | --- |
| SM_WT | GU257342  GU257335  GU257349  JQ397380  JQ397385  JQ397372  GU257354  GU257346  GU257352  GU257356  GU257336  JQ082122  JQ397388  GU257345  GU257348  JQ397383  JQ397373  JQ397379  JQ397399  GU257360  GU257338  JQ397398  JQ397386  JQ397374  JQ397384  GU257334  JQ397381  JQ397393  JQ397394  JQ397395  JQ397396 | Zan 9  Zan 2  Zan 16  CK2b  CK3c  CK1b  Zan 21  Zan 13  Zan 19  Zan 23  Zan 3  Mafia2  TA1b  Zan 12  Zan 15  CK3a  CK1c  CK2a  MD1  Zan 27  Zan 5  MU1  CK3d  CK1d  CK3b  Zan 1  CK2c  ZA1  ZA2  ZA3  ZA4 | Zanzibar: Unguja  Zanzibar: Unguja  Zanzibar: Unguja  Coastal Kenya: Kinango  Coastal Kenya: Nimbodze  Coastal Kenya: Rekeke  Zanzibar: Unguja  Zanzibar: Unguja  Zanzibar: Unguja  Zanzibar: Unguja  Zanzibar: Unguja  Tanzania: Mafia  Tanzania: Mwanza  Zanzibar: Unguja  Zanzibar: Unguja  Coastal Kenya: Nimbodze  Coastal Kenya: Rekeke  Coastal Kenya: Kinango  Madagascar  Zanzibar: Unguja  Zanzibar: Unguja  Mauritius: Vallee Pitot  Coastal Kenya: Nimbodze  Coastal Kenya: Rekeke  Coastal Kenya: Nimbodze  Zanzibar: Unguja  Coastal Kenya: Kinango  Zambia: Simunjalala  Zambia: Katunga  Zambia: Kafue  Zambia: Lisiko | [5]  [5]  [5]  [14]  [14]  [14]  [5]  [5]  [5]  [5]  [5]  [15]  [14]  [5]  [5]  [14]  [14]  [14]  [14]  [5]  [5]  [14]  [14]  [14]  [14]  [5]  [5]  [14]  [14]  [14]  [14] |
| Subset 1 | GU257358 | Zan 25 | Zanzibar: Unguja | [5] |
| Subset 2 | JQ082121  GU257339  GU257340  GU257344 | Mafia1  Zan 6  Zan 7  Zan 11 | Tanzania: Mafia  Zanzibar: Unguja  Zanzibar: Unguja  Zanzibar: Unguja | [15]  [5]  [5]  [5] |
| Subset 3 | JQ397377  JQ397376  JQ397382  JQ397375  JQ397378  JQ397371  GU257353  GU257350  GU257359  GU257355  JQ397368  GU257343  JQ397391  JQ397339  JQ397397  JQ397337  JQ397355  JQ397341  JQ397363  JQ397332  JQ595405  JQ397342  JQ397344  JQ397349  JQ397356  JQ397357  JQ357358  JQ397359  JQ397360  JQ397361  JQ397362  JQ397347  GU257341  GU257347  GU257351  GU257357  JQ397330  JQ397331  JQ397333  JQ397334  JQ397335  JQ397336  JQ397338  JQ397340  JQ397343  JQ397345  JQ397346  JQ397348  JQ397350  JQ397351  JQ397352  JQ397353  JQ397354  JQ397364  JQ397365  JQ397366  JQ397367  JQ397369  JQ397370  JQ397387  GU257337  JQ397389  JQ397390  JQ397392 | CK1g  CK1f  CK2d  CK1e  CK1h  CK1a  Zan 20  Zan 17  Zan 26  Zan 22  EG1  Zan 10  MW2b  SE7b  SA1  SE6b  CA1b  SE8b  CA1j  SE2b  SE3b  SE8c  MA1  GA1  CA1c  CA1d  CA1e  CA1f  CA1g  CA1h  CA1i  NI1b  Zan 8  Zan 14  Zan 18  Zan 24  SE1  SE2a  SE3a  SE4  SE5  SE6a  SE7a  SE8a  SE9  MA2  NI1a  NI2  LB1  GB1  NG1  CA1  CA1a  CA2  CA3  CA4  CA5  SU1  KE2  TA1a  Zan 4  MW1  MW2a  MW3 | Coastal Kenya: Rekeke  Coastal Kenya: Rekeke  Coastal Kenya: Kinango  Coastal Kenya: Rekeke  Coastal Kenya: Rekeke  Coastal Kenya: Rekeke  Zanzibar: Unguja  Zanzibar: Unguja  Zanzibar: Unguja  Zanzibar: Unguja  Egypt  Zanzibar: Unguja  Malawi: Likoma  Senegal: Kolda  South Africa: Durban  Senegal: Tambacounda  Cameroon: Bessoum  Senegal: Barkedji  Cameroon: Bessoum  Senegal: Mbodiene  Senegal: Nder  Senegal: Barkedji  Mali: Niger Delta  Gambia  Cameroon: Bessoum  Cameroon: Bessoum  Cameroon: Bessoum  Cameroon: Bessoum  Cameroon: Bessoum  Cameroon: Bessoum  Cameroon: Bessoum  Niger: Libore  Zanzibar: Unguja  Zanzibar: Unguja  Zanzibar: Unguja  Zanzibar: Unguja  Senegal: Guédé-Chantier  Senegal: Mbodiene  Senegal: Nder  Senegal: Temeye  Senegal: Podor  Senegal: Tambacounda  Senegal: Kolda  Senegal: Barkedji  Senegal  Mali: Niger Delta  Niger: Libore  Niger: Falmado  Liberia: Wenshu  Guinea Bissau: Gabu  Nigeria: Kanu  Cameroon: Bessoum  Cameroon: Bessoum  Cameroon: Okuro  Cameroon: Loum  Cameroon: Barombi Mbo  Cameroon: Barombi Kotto  Sudan: Gezira  Kenya: Taveta  Tanzania: Mwanza  Zanzibar: Unguja  Malawi: Chembe  Malawi: Likoma  Malawi | [14]  [14]  [14]  [14]  [14]  [14]  [5]  [5]  [5]  [5]  [14]  [5]  [14]  [14]  [14]  [14]  [14]  [14]  [14]  [14]  [14]  [14]  [14]  [14]  [14]  [14]  [14]  [14]  [14]  [14]  [14]  [14]  [5]  [5]  [5]  [5]  [14]  [14]  [14]  [14]  [14]  [14]  [14]  [14]  [14]  [14]  [14]  [14]  [14]  [14]  [14]  [14]  [14]  [14]  [14]  [14]  [14]  [14]  [14]  [14]  [5]  [14]  [14]  [14] |

**References**

1. Edgar RC. MUSCLE: multiple sequence alignment with high accuracy and high throughput. Nucleic Acids Research 2004;32. <https://doi.org/10.1093/nar/gkh340>.

2. Ying Z-M, Tu B, Liu L, Tang H, Tang L-J, Jiang J-H. Spinach-based fluorescent light-up biosensors for multiplexed and label-free detection of microRNAs. Chemical Communications 2018;54. <https://doi.org/10.1039/C8CC00123E>.

3. Lockyer AE, Olson PD, Østergaard P, Rollinson D, Johnston DA, Attwood SW, et al. The phylogeny of the Schistosomatidae based on three genes with emphasis on the interrelationships of *Schistosoma* Weinland, 1858. Parasitology 2003;126. <https://doi.org/10.1017/S0031182002002792>.

4. Kane RA, Southgate VR, Rollinson D, Littlewood DTJ, Lockyer AE, Pags JR, et al. A phylogeny based on three mitochondrial genes supports the division of Schistosoma intercalatum into two separate species. Parasitology 2003;127. <https://doi.org/10.1017/S0031182003003421>.

5. Webster BL, Culverwell CL, Khamis IS, Mohammed KA, Rollinson D, Stothard JR. DNA barcoding of Schistosoma haematobium on Zanzibar reveals substantial genetic diversity and two major phylogenetic groups. Acta Tropica 2013;128. <https://doi.org/10.1016/j.actatropica.2012.06.002>.

6. Djuikwo-Teukeng FF, Simo AK, Allienne J-F, Rey O, Njayou Ngapagna A, Tchuem-Tchuente LA, et al. Population genetic structure of Schistosoma bovis in Cameroon. Parasites & Vectors 2019;12. https://doi.org/10.1186/s13071-019-3307-0.

7. Hanelt B, Brant S, Steinauer ML, Maina GM, Kinuthia JM, Agola LE, et al. *Schistosoma kisumuensis* n. sp. (Digenea: Schistosomatidae) from murid rodents in the Lake Victoria Basin, Kenya and its phylogenetic position within the *S. haematobium* species group. Parasitology 2009;136. <https://doi.org/10.1017/S003118200900643X>.

8. Attwood SW, Fatih FA, Upatham ES. DNA-Sequence Variation Among Schistosoma mekongi Populations and Related Taxa; Phylogeography and the Current Distribution of Asian Schistosomiasis. PLoS Neglected Tropical Diseases 2008;2. <https://doi.org/10.1371/journal.pntd.0000200>.

9. Catalano S, Léger E, Fall CB, Borlase A, Diop SD, Berger D, et al. Multihost Transmission of *Schistosoma mansoni* in Senegal, 2015–2018. Emerging Infectious Diseases 2020;26. <https://doi.org/10.3201/eid2606.200107>.

10. Mouahid G, Nguema RM, Al Mashikhi KM, Al Yafae SA, Idris MA, Moné H. Host‐parasite life‐histories of the diurnal *vs* . nocturnal chronotypes of *Schistosoma mansoni* : adaptive significance. Tropical Medicine & International Health 2019;24:692–700. <https://doi.org/10.1111/tmi.13227>.

11. Catalano S, Sène M, Diouf ND, Fall CB, Borlase A, Léger E, et al. Rodents as Natural Hosts of Zoonotic Schistosoma Species and Hybrids: An Epidemiological and Evolutionary Perspective From West Africa. The Journal of Infectious Diseases 2018;218:429–33. <https://doi.org/10.1093/infdis/jiy029>.

12. Schols R, Mudavanhu A, Carolus H, Hammoud C, Muzarabani KC, Barson M, et al. Exposing the Barcoding Void: An Integrative Approach to Study Snail-Borne Parasites in a One Health Context. Frontiers in Veterinary Science 2020;7. <https://doi.org/10.3389/fvets.2020.605280>.

13. Brant SV, Fairet E, Shirley MH, Petrželková KJ, Modrý D, Červená B. Schistosoma mansoni in Gabon: Emerging or Ignored? The American Journal of Tropical Medicine and Hygiene 2016;95:849–51. <https://doi.org/10.4269/ajtmh.16-0446>.

14. Webster BL, Emery AM, Webster JP, Gouvras A, Garba A, Diaw O, et al. Genetic Diversity within Schistosoma haematobium: DNA Barcoding Reveals Two Distinct Groups. PLoS Neglected Tropical Diseases 2012;6:e1882. <https://doi.org/10.1371/journal.pntd.0001882>.

15. Stothard JR, Ameri H, Khamis IS, Blair L, Nyandindi US, Kane RA, et al. Parasitological and malacological surveys reveal urogenital schistosomiasis on Mafia Island, Tanzania to be an imported infection. Acta Tropica 2013;128:326–33. <https://doi.org/10.1016/j.actatropica.2012.09.006>.
