# Supplementary material for: Specific Nucleic AcId Ligation for the detection of Schistosomes: SNAILS
Source: PLoS Negl Trop Dis. 2022 Jul 26;16(7):e0010632. doi: 10.1371/journal.pntd.0010632 (PMC9355235; doi:10.1371/journal.pntd.0010632)
Supplement: Attachment — Submitted filename: Webb_etal_SNAILS-response to reviewer.docx [file pntd.0010632.s003.docx]

Co-director of Centre for Synthetic Biology and Innovation & UK Innovation and Knowledge

Centre for Synthetic Biology (SynbiCITE)

Imperial College London

Head of Section of Structural and Synthetic Biology

Department of Infectious Disease

Sir Alexander Fleming Building

South Kensington Campus

Exhibition Road

London, SW7 2AZ, UK

Tel: +44 (0) 207 594 5327

p.freemont@imperial.ac.uk

4^th^ May 2022

Dear Editors,

Re: “**Specific Nucleic AcId Ligation for the detection of Schistosomes: SNAILS**”- Webb, Allan and Kelwick *et al.* **Ref PNTD-D-21-01756**

We would like to thank the editor of PLoS NTD and the reviewers for their constructive comments. To address the points raised, we have modified the text of the manuscript accordingly. Below, we provide a point-by-point response to each comment.

We look forward to receiving a positive response.

Yours faithfully,

Professor Paul Freemont

**Editor and Reviewer comments:**

**Reviewer #1: The objective of the study is to demonstrate proof-of-concept for a DNA-based biosensor for species-specific detection of i) synthetic DNA targets; and ii) field-collected samples, from two species of schistosome that cause human health problems. This is testable and the study design is appropriate to address the stated objectives for the synthetic targets derived from reference sequences as the authors use a total of nine closely related species (some with multiple sequenced genomes) to ensure specificity and they go on to extensively optimise a number of relevant parameters.**

We thank the reviewer for their positive comments that the study design is appropriate to address the stated objectives.

**However, the use of only two adult worms of each species collected in the field is a concern as one of the two S. haematobium already had two base changes in a probe target region. There is no discussion of the potential or measured rate of variation in nature at those key base positions. They artificially mutated a range of single, double or multiple base changes to the S. mansoni wild-type target region to demonstrate that the probes retained specificity but this strand could be tied more clearly to robustness against likely natural variation.**

**It is also unclear why DNA from only two organisms for each species was available for the study as this is not a large sample size. Without further understanding of natural sequence variation it is difficult to conclude that the probes would be specific in a large scale study of samples from nature so I would expect to see this addressed in the discussion.**

We have carried out additional bioinformatic analysis on a range of publicly available sequences (see Supplementary Tables 6 & 7) and identified natural subset variations in the target sequences of *S. mansoni*-specific probe set 5 and *S. haematobium*-specific probe set 1. Furthermore, we have tested a panel of 6 more *S. mansoni* gDNA samples and these data have been added to the results section (see Fig. 6 e, f, g & h), where we also add additional text on natural *Schistosoma* intraspecies sequence variations.

**The statistical methods deployed are appropriate to support the conclusions. There are no concerns around ethical or regulatory requirements.**

**Results

Reviewer #1: The analysis presented matched the analysis plan and results are clearly and completely presented but may be too complete in that some of the information in the captions is easily discoverable in the methods so need not be repeated in each figure.**
**For example, most figures contain “Fluorescence outputs of reactions were analysed using a CLARIOstar plate reader (440-15 nm/510-20 nm; Gain 1500).” This information is already presented in the methods and could be removed from the captions. Likewise, “Mean and Standard error of the mean calculated using GraphPad Prism 9.1.1.” In the supplementary table captions is redundant to the methods section.**

We have amended the figure legends to make them more concise.

**I request that the authors include a reference to the supplementary information in Fig 2,3,5 and 6 captions where their probe sequence data is included as a non-copyable image, so the reader knows that they can find a text version elsewhere.**

We have added this reference to the supplementary information in the figure legends as requested.

**Supplementary Figure 9, 10, 16, 17 are not high resolution in the PDF provided.**

We thank the reviewer for highlighting this, and we have provided higher resolution versions of these figures.

**The PDF contains a very large number of tables with hourly plate reader date and calculations. This makes it difficult to find the other information that the reader is more likely to need, like the tables of nucleic acid sequences. Given this and that PLOS recommends that spreadsheets are preferable to PDFs or images when providing tabulated data, I would suggest providing a spreadsheet containing all of this experimental data (e.g. one experiment per sheet) rather than a PDF table, leaving the PDF shorter and containing only figures and resource tables.**

As suggested, we have removed the tables with the hourly plate reader data and have moved the information to a new Supplementary Spreadsheet file. Each tab on the spreadsheet is listed with the figure that data corresponds to i.e. Fig 1 c.

**Conclusions**

**Reviewer #1: The conclusion is that the biosensor can detect and differentiate between two human health relevant schistosomes, S. mansoni and S. haematobium, as well as discriminate these from other closely related species such as S. bovis and S. rodhaini and that ‘SNAILS’ probes are amenable to rapid design iterations. This is supported by the data presented but I would further discuss the very limited sample size for field-collected samples and describe the level of intraspecies sequence variation that exists (if known) or may potentially exist, given one of the four samples tested had two base pairs variance. The rapid design iterations will mitigate against this causing issues during the design phase but not in field use if the natural population has high variance – for this I would expect to see a much larger sample size. The public health relevance of the study is described in terms of being an environmental surveillance tool to monitor presence of the schistosomes and the effectiveness of control measures.**
**I would also encourage some discussion of future developments which would be needed to advance the biosensor design as the introduction states that the authors concentrated "on the need for a species-specific point-of-care (PoC)/point-of-test (PoT) diagnostic" but there is then no further mention of the PoT potential for this assay, which currently uses PCR, one of the harder amplification methods to convert to PoT, followed by a further enzymatic step and then addition of the probe and ligase. It is also unclear if the fluorescent output is easy to read without a real-time system. These could be limitations for a PoT approach.

SNAILS is a redesign of the spinach aptamer assay for ssDNA detection and applications of this to other targets could be mentioned in the discussion alongside any planned further research.**

To address these comments, we have carried out further bioinformatic analysis on a range of publicly available sequences (see Supplementary Tables 6 & 7). We identified that there were natural subset sequence variations for the target sequences. We also tested the probes against 6 more *S. mansoni* gDNA samples from worms isolated from disparate geographical locations, some of which did have these subset sequence variations. These data are presented in the results section (Fig 6 e, f, g & h). For this proof-of-concept study, we were able to detect and differentiate several of these new samples. As the reviewer mentions, the advantage of our biosensor is the rapid design iterations that can be achieved. As more nucleotide sequences become publicly available, further bioinformatic analysis will enable validation of new biosensor probe designs.

We have also significantly revised the discussion section of the manuscript to discuss the potential longer-term applications of our ‘SNAILS’ biosensor including other potential targets, possible future optimisations such as reducing costs further, and the use of other aptamers to enable assay multiplexing.

**Editorial and Data Presentation Modifications?**

**Reviewer #1: Lines 207-250 belong in the introduction as they provide further detail about existing control measures and the landscape of existing molecular diagnostics into which this project fits, adding context and situating it within the literature. I would include content from line 250 onwards as discussion and add some further detail on natural sequence variation in populations and its potential effect on the assay specificity.**

As suggested, we have moved these sections to the introduction, and have re-focussed the discussion to highlight future applications of the biosensor and other important aspects recommended by the reviewer.

**L.244 the comment that sequencing is expensive should be put in context and prefaced by a comment that the previous techniques would typically need to be followed by sequencing to enable species level identification.**

Thank you, we have made the recommended changes.

**There are a number of typos in the text but they all look like they should be detected by spell check so I haven’t listed them.**

We have proof read the revised manuscript and have made appropriate corrections.

**Summary and General Comments**

**Reviewer #1: Overall this is a good lab-level proof of concept for a diagnostic biosensor design with good potential for impacting surveillance efforts in schistosomiasis. The design is a novel and original adaptation of an existing assay to detect ssDNA instead of RNA which may be of interest to other biosensor designers. It is intended to eventually be PoT but the proof of concept was performed with standard lab instrumentation and multiple steps in the lab with no further information provided on PoT potential, which makes it less relevant in its current form to a wide audience of practitioners.**

To address these comments, we have significantly revised the discussion section of the manuscript to provide additional discussion on potential future work to optimise the ‘SNAILS’ biosensor assay further (i.e. reducing assay costs and adding multiplexing capabilities) and how we envision progressing, beyond our current proof-of-concept study, towards future point-of-care applications.

**Being species-specific without the need for sequencing is a compelling alternative to existing assays that require sequencing to provide that level of specificity. The major weakness of the study is the aforementioned small sample size for organismal DNA and lack of clarity on intraspecies sequence variation in the field which means it falls short of demonstrating broader application. However, this is consistent with the claims of the authors, who clearly describe it as a proof of concept. The experiments appear to have been well executed and data is provided in full. I have no further comments on the data presented and its analysis and interpretation**.

We thank the reviewer for their comments. As mentioned above we have added additional bioinformatic analyses of publicly available DNA sequences to provide additional information on natural sequence variation in the target region of the probes. We have also tested 6 more additional *Schistosoma* gDNA samples from worms isolated from disparate geographical locations.

**Demonstrating successful detection across a much wider set of field samples would improve the paper's importance and relevance to researchers, practitioners, or policy makers in the field of NTDs. This could involve further collection of field samples if these are not already available to the authors, which could take significant amounts of time. The paper stands alone in terms of the biosensor design so could be published in another journal with different criteria with quite minor revisions, particularly recognising limitations in the discussion section, but I feel this major revision would substantially improve its fit with the PLOS NTD criteria.**

We thank the reviewer for their advice and recommendations. In response we have significantly revised the manuscript adding new bioinformatic and experimental data. This includes the testing of additional field samples of *S. mansoni*.

**An exception would be if the authors can convincingly demonstrate without additional experimental work (e.g. through presentation of secondary data from schistosome genetic/genomic studies or other means) that the likelihood of sequence mutations across a broad population within each species is low enough that the lab results obtained with synthetic DNA from reference genomes is representative of likely performance in the field. It is possible that this data exists but was not presented clearly in the paper. They did artificially mutate a range of single, double or multiple base changes to the S. mansoni wild-type target region to demonstrate specificity and may consider this is sufficient evidence that natural variation will not perturb the function of the assay. Discussing this in more detail and why it is likely a good proxy for natural variation could also be sufficient.**

We have carried out additional bioinformatic analyses to address the natural intraspecies sequence variation in schistosomes. Some of the newly tested *Schistosoma* samples matched some of the identified sequence variation subsets listed in Supplementary Table 6. These data have been added to the manuscript and we feel that the ‘SNAILS’ biosensor technology, as indicated by the reviewer, is amenable to rapid design iterations. This could enable further probes to be designed in future studies to further improve intraspecies and interspecies discrimination and detection.
